# Advanced Chlorine Photolysis for Carbamazepine Degradation: Tuning Radicals for Dissolved Organics

**DOI:** 10.1021/acsestwater.5c01498

**Published:** 2026-04-14

**Authors:** Caleb Osei-Appau, Madhusudan Kamat, Eddy Petit, Jeanne Le Beux, Benoit Teychené, Volodymyr V. Tarabara, François Zaviska, Geoffroy Lesage, Samuel D. Snow

**Affiliations:** † Department of Civil and Environmental Engineering, 5779 Louisiana State University, 3255 Patrick Taylor Hall, Baton Rouge, Louisiana 70803, United States; ‡ UAR Plateforme d’Analyses et Caractérisations (PAC) Chimie Balard Montpellier, Univ Montpellier, CNRS, ENSCM, Place Eugène Bataillon, 34095 Montpellier, France; § IEM (Institut Européen des Membranes), UMR 5635 (CNRS-ENSCM-UM), Université de Montpellier, Place E. Bataillon, 34095 Montpellier, France; ∥ IC2MP (Institut de Chimie des Milieux et Matériaux de Poitiers), UMR CNRS 7285), Université de Poitiers, 1 rue Marcel Doré, 86073 Poitiers Cedex 9, France; ⊥ Department of Civil and Environmental Engineering, Michigan State University, 428 S. Shaw Lane, East Lansing, Michigan 48824, United States; # Center for European and Eurasian Studies, International Studies and Programs, Michigan State University, East Lansing, Michigan 48824, United States

**Keywords:** Carbamazepine, UV/chlorine Advanced Oxidation Process, Hypochlorite, O_3_, Dissolved Organic Matter (DOM), Electron Paramagnetic Resonance, Microtoxicity, Degradation Byproducts

## Abstract

The use of advanced oxidation processes (AOPs) for micropollutant removal in wastewater is challenging, given the scavenging effects of dissolved organic matter (DOM). Chlorine photolysis (UV/Cl) offers unique advantages over conventional AOPs, with wavelength- and pH-dependent generation of reactive species, while being largely compatible with existing disinfection dosing infrastructure. Here, the UV/Cl treatment of carbamazepine (CBZ) was optimized as a test case by varying UV wavelength and solution pH in the presence of humic acid and various types of wastewater DOM isolates (colloidal, hydrophobic, and transphilic). CBZ degradation rates, competitive quenching experiments, and electron paramagnetic resonance spectroscopy revealed important wavelength- and pH-dependent trade-offs in the dominant oxidants. At pH 3, CBZ degradation was hindered by DOM due to scavenging of nonselective radicals. At pH 8, ozone formation minimized DOM interference and improved CBZ removal. Shifting to longer wavelengths caused changes parallel to raising the pH. Three-dimensional excitation–emission matrix fluorescence analysis revealed preferential reactivity with fulvic- and humic-like fluorophores during treatment. Microtoxicity bioassays indicated a slight increase in acute toxicity after UV/Cl exposure, and LC–MS identified hydroxylated, epoxidized, and chlorinated transformation products. Results highlight the potential for strategic optimization of UV/Cl to mitigate DOM interference in water treatment.

## Graphical abstract

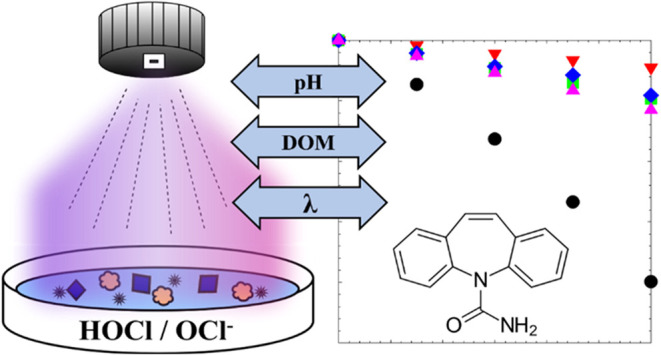

## Introduction

Emerging contaminants including pharmaceutical and personal care products (PPCPs) have become a significant subject of interest over the past decades.^1–3^ PPCPs comprise a broad spectrum of organic compounds that pose a range of potential risks of adverse environmental and health effects, including endocrine disruption, aquatic toxicity, genotoxicity, and development of resistance in pathogenic bacteria.^2,4,5^ Recent research has revealed that various PPCPs are difficult to remove by conventional physical, biological, and chemical water treatment techniques including sedimentation, coagulation, and flocculation.^4,6^ Among these pharmaceuticals, carbamazepine (CBZ) is a particularly refractory micropollutant of significant environmental concern. CBZ is resistant to biodegradation and is not effectively removed by conventional water treatment methods. Some studies have reported the presence of CBZ in wastewaters (≤6.3 μg/L), in surface waters (≤1.1 μg/L) and in drinking water (≤0.03 μg/L).^7^ Advanced oxidation processes (AOPs) are increasingly employed by water treatment facilities to reduce the concentrations of pharmaceuticals.^3,8–10^ AOPs offer a potential solution to the problems posed by PPCPs, but certain constituents of water limit the applicability of AOPs, especially in wastewaters.^1,11–20^

The presence of dissolved organic matter (DOM) poses an important challenge to the application of AOPs to natural and wastewaters, because nontarget constituents compete for the reactive species produced.^21^ The heterogeneous and ubiquitous nature of DOM^22–24^ complicates efforts to understand and mitigate their quenching effects on AOPs. For example, wastewater DOM encompasses a wide range of complex organic molecules (e.g., nucleic acids, polysaccharides, and humic substances) that are known to rapidly quench ^•^OH, the most common radical agent in many AOPs.^25–28^ Novel AOP systems offer a promising advantage of a wider array of reactive species so that unwanted quenching can be avoided. The advent of far-UV LEDs allows particularly interesting advancements in chlorine photolysis (UV/Cl), because the type of reactive species generated during UV/Cl is wavelength-dependent.^29^ The UV/Cl system produces a range of reactive species (^•^OH, Cl^•^, Cl_2_^•–^, O_3_) from the UV activation of the HOCl/OCl^–^ system.^30–35^ These reactive species exhibit different reactivities with targeted pollutants and nontarget cosolutes such as DOM, carbonates, and other inhibitory compounds.^31,36^ The collection of radicals generated by the UV/Cl AOP provides numerous opportunities to overcome the challenges of nontarget reactions with DOM.^32^ Among these, hydroxyl radicals (^•^OH), being the least selective, are quenched rapidly by these inhibitors.^31^ Chlorine radicals (Cl^•^) are also highly reactive, but these radicals are more selective than ^•^OH, giving Cl^•^ a slight advantage over ^•^OH radicals,^32^ especially in the presence of DOM.^32^ In the presence of Cl^–^, Cl^•^ exists in equilibrium with Cl_2_^•–^, which is more selective than both ^•^OH and Cl^•^. During OCl^–^ photolysis, O_3_ is formed and can be a primary reactive oxygen species (ROS) end point.^32^ The use of O_3_ as a selective oxidant (and a UV-AOP substrate) is well documented.^32^ While the impact of DOM on AOPs has been well-researched, very few have studied the role of disparate DOM types on the UV/Cl system.^22,37^

UV/Cl stands out as a promising AOP that presents an attractive alternative to conventional AOPs due to its affordability, added control over ROS and reactive chlorine species (RCS) outcomes and applicability in natural or waste-waters.^1,38–41^ One study showed that the UV/Cl process can efficiently degrade a range of micropollutants.^38^ Factors such as pH, wavelength, and chlorine concentration significantly influence its effectiveness.^20,39,41,42^ Under controlled laboratory and tertiary-effluent conditions with limited matrix interference, the UV/Cl process has been reported to achieve electrical energy per order (*E*_EO_) values below 1 kWh/m^3^/order for micropollutants such as carbamazepine, nitrobenzene, benzotriazole, and methyl orange.^39,43,44^ Here, the UV/Cl process is explored primarily in the context of a tertiary polishing step following biological wastewater treatment, particularly for reclamation and reuse where recalcitrant micropollutants such as CBZ persist.^20,36^ More broadly, UV/Cl may find excellent application in potable water treatment from point-of-use to municipal scale, leveraging and adding to existing UV or chlorine dosing disinfection infrastructure to simultaneously achieve disinfection and oxidation with modest additional energy input.^20,36,45^ Comparative studies have shown that UV/Cl can be more energy-efficient in degrading micropollutants compared to other AOPs.^43,46^ Most previous studies of chlorine photolysis and other AOPs have been conducted using low or medium-pressure mercury lamps as UV sources.^3,9,10^ UV-LEDs offer a promising alternative for driving the UV/Cl process, providing a wide range of wavelengths to optimize the production of desired reactive species.^9^

The combination of UV-LEDs and chlorine offers an attractive approach for degrading recalcitrant contaminants like CBZ. However, inhibitory reactions by DOM remain a largely unexplored challenge. Here, we explore the utility of UV-LEDs to drive the UV/Cl process to overcome these unwanted quenching reactions—common to AOPs. Electron paramagnetic resonance (EPR) spectroscopy is employed to directly detect and quantify the formation of ROS and RCS during UV/Cl photolysis. The degradation of CBZ is further evaluated through the identification of key degradation byproducts, including hydroxylated, epoxidized, and chlorinated intermediates, to elucidate dominant oxidation pathways and assess the role of selective oxidants. Finally, Microtoxicity bioassays using *Vibrio fischeri* are performed to assess acute toxicity evolution during CBZ degradation, enabling a correlation between radical chemistry, byproduct formation, and resulting ecotoxicological impacts. Here, the ROS and RCS generation is tuned in the chlorine photolysis system to optimize the destruction of CBZ in the presence of fractionated wastewater DOM isolates.

## Materials and Methods

### Materials and Chemicals

Sodium hypochlorite from Alfa Aesar (8–10% available chlorine) was used as a source of free chlorine. CBZ and humic acid (HA) were also obtained from Alfa Aesar. All solvents used were high-performance liquid chromatography (HPLC) grade. Nanopure water was used for all solutions. O_3_ concentrations were measured using HACH O_3_ AccuVac ampules for low-range (0.01 to 0.25 mg/L) O_3_ concentrations.

### Photochemical Experiments

Photochemical experiments were conducted using a magnetically stirred photoreactor in an enclosed UV cabinet. The light sources used for the experiments were UV–B (LG Innotek 6868 UV_305_ LED) and UV–C (Sankyo Denki Co., Japan, 15 W low-pressure mercury lamp) with emission peaks at 305 and 254 nm, respectively. The distance between the reaction vessel and light source was 20 cm. The irradiance at the location of the vessel for the experiments was measured as 458 μJ/cm^2^ and 195 μJ/cm^2^ using a UVX Digital Radiometer with UVX-25 and UVX-31 sensor probes (Analytik Jena, San Gabriel, California), respectively. A 15 mL vessel was used for the experiments and different concentrations of DOM fractions, chlorine, and target pollutants were added accordingly to study the efficacy of the UV/Cl process. The pollutant concentrations were measured periodically via an Agilent 1260 Infinity HPLC system. Fluence calculations were used to find the observed photodegradation rate constants (*k*_obs_) and the difference was used to compare the inhibitory effect of the fractions. The degradation rates were assumed to be pseudo-first order for analyses.

A series of selective quenching experiments were conducted to identify the dominant reactive species responsible for CBZ degradation under different UV/Cl conditions. These experiments were designed to discern the relative contribution of each radical pathway within the system. Tert-butyl alcohol (TBA > 95%, Thermo Fisher Scientific) and nitrobenzene (NBZ, 99%, Thermo Fisher Scientific) were used as a selective scavengers for •OH; sodium benzoate (SBZ, >99%, Sigma-Aldrich) was used to quench Cl^•^; and sodium azide (SAZ, 99%, Thermo Fisher Scientific) was used as a known ^1^O_2_ quencher.

### DOM Characterization

DOM isolates from the bulk supernatant of a membrane bioreactor (MBR)-based wastewater treatment plant in La Grande-Motte, France were used here. The DOM fractionation was described previously, yielding hydrophobic (HPO) and transphillic (TPI) fractions.^24^ DOM fractions were characterized via three-dimensional excitation–emission matrix (3DEEM) fluorescence spectroscopy before and after exposure to UV/Cl treatment. Fluorescence spectra were grouped by spectral regions. According to Chen et al., the fluorescence spectra can be divided into spectral regions, which correspond to fluorophores of known types;^47^ additional details can be found in Text S1 and in Table S1 of the Supporting Information (SI). In this categorization, zones I through V are associated with specific types of fluorophores, namely (**I**) aromatic proteins-like, type I; (**II**) aromatic proteins-like, type II; (**III**) fulvic-like; (**IV**) soluble microbial products (SMP)-like; and (**V**) humic-like fluorophores.

### Microtoxicity Assays

The bacterial strain *Vibrio fischeri* NRRL B11177, obtained from Hach Lange GmbH (Germany), was used to evaluate the acute toxicity of CBZ and its degradation byproducts. The assay followed the standard Microtox protocol (Modern Water Inc., UK), which measures the inhibition of bacterial bioluminescence as an indicator of sample toxicity. Test solutions were prepared using an osmotic-adjusting solution (Milli-Q water with 22% NaCl) and a 2% NaCl diluent, with all samples diluted to 45% of their initial concentration to maintain optimal bacterial activity. Luminescence was recorded after 5 and 15 min of exposure, and inhibition (%) was calculated using the corrected response ratio *R*(*t*) derived from control solutions to account for natural signal decay.^48,49^

### CBZ Intermediates Analysis

The chromatographic separation was performed on a Waters Atlantis Premier BEH C18 AX column (50 mm × 2.1 mm, 1.7 μm) at 25 °C, using a Thermo Scientific Vanquish UHPLC system. The mobile phase consisted of (A) water with 0.1% formic acid and (B) acetonitrile with 0.1% formic acid. The flow rate was 0.5 mL/min with a 5 min gradient. The gradient profile was as follows: 0% B for 1 min, increased to 100% B over 2 min, held at 100% B until 3.5 min, and then rapidly re-equilibrated to 0% B until the end of the 5 min run. The injection volume was 10 μL. High-resolution mass spectrometry analysis was carried out using a Thermo Scientific Orbitrap ID-X Tribrid mass spectrometer with a heated electrospray ionization (H-ESI) source. The instrument was operated in both positive and negative ionization modes.

### EPR Measurements

The following spin trap reagents and standards were used for electron paramagnetic resonance (EPR) analysis without further purification: 5,5-dimethyl-1-pyrroline-N-oxide (DMPO, >99%; Thermo Fisher Scientific), 5-*tert*-Butoxycarbonyl-5-methyl-1-pyrroline-N-oxide (BMPO, 97%; Ambeed Inc.), α-phenyl *N*-tertiary-butyl nitrone (PBN; Dojindo). A Bruker Magnettech ESR5000 spectrometer (Bruker Corporation, Billerica, Massachusetts) equipped with a high-sensitivity resonator was used in a closed-system configuration with direct UV exposure to the EPR tube during data acquisition. This in situ setup allowed for the independent measurement of DMPO or BMPO radical adduct formation without delay, ensuring reliable, real-time monitoring of the UV/Cl system. The radical adduct signals and hyperfine coupling constants of *a*, β, and γ were obtained by fitting spectra using ESR Studio software. All scans were conducted in kinetic series at room temperature with the following EPR instrument parameters: power of 10.0 mW, modulation amplitude of 0.1 mT, modulation frequency of 100 kHz, sweep time of 20 s, coupling time of 10.00 s, sweep width of 200 mT, microwave attenuation of 10 dB, and center field of 336 mT in the UV/Cl system.

## Results and Discussion

### EPR Analyses of the UV/Cl System

EPR spectroscopy was employed to quantify the production of ROS and RCS formed during UV_305_/Cl under different pH conditions. The formation of spin-trapped adducts are plotted in Figure 1, along with the corresponding EPR spectra for BMPO–OH and DMPO-X. Adjustments to pH corresponded with distinct changes in radical adduct formation rates, as measured by the growth of EPR signal intensities over time. The rate constants for each experiment are reported in Table S2 of the SI. Figure 1A shows the time-resolved accumulation of BMPO–OH adducts (Figure S1), the result of reactions between BMPO and ^•^OH, with the characteristic quartet pattern^50^ of BMPO–OH shown at 10, 30, and 60 min (Figure 1B). Hydroxyl radical adduct generation was fastest under acidic conditions (pH 3), while pH 10 produced the lowest yields. Similarly, Figure 1(C,D) presents the formation of DMPO-X adducts, which was strongly pH-dependent, with the highest concentrations at pH 3 and progressively lower levels with increasing pH. These observations match other reports that also showed that acidic conditions favor ^•^OH formation during UV/Cl processes.^29,41^ Previous studies attributed the DMPO-X signal to an oxidized derivative formed through the reaction of DMPO–OH additional radicals.^51,52^ In other words, DMPO-X is an oxidized derivative of DMPO–OH, formed through DMPO–OH’s reaction with ^•^OH and Cl^•^, and perhaps with Cl_2_^•–^.^51–53^ The EPR spectra corresponding to DMPO-X (Figure 1D) showed the characteristic seven-line splitting pattern of DMPO-X.^51,52^ Previous studies have had difficulty confirming the direct trapping of Cl^•^ using in H_2_O. DMPO does react with Cl^•^ to form a DMPO-Cl adduct; however, this intermediate is highly unstable in water, undergoing rapid hydrolysis to DMPO–OH and subsequent overoxidation to the DMPO-X derivative.^51,52^ This instability makes direct quantification of Cl^•^ formation by EPR difficult, but the formation of DMPO-X may provide some insight into Cl^•^ prevalence. Recently, Wang et al. showed that hydrolysis of DMPO-Cl in ^17^O-enriched water accounts for about 59% of DMPO–OH observed in a UV/PtCl_6_^2–^ system, which produces strictly Cl^•^; they also found that in water-free organic solvents, DMPO–OH was not formed.^54^ It may be possible to approximate a steady-state concentration of Cl^•^ with more information about Cl^•^’s reactivity with DMPO–OH to form DMPO-X. Such a method would need to account for [^•^OH] also, because DMPO-X can be formed via several routes that overoxidize the DMPO–OH adduct.^55,56^ Here, the formation kinetics of BMPO–OH and DMPO-X and the respective molar concentrations present after an hour of UV_305_/Cl are summarized in Tables S2 and S3 of the SI for different pH values. The DMPO-X and BMPO–OH trends exhibited a strong pH dependence, aligning with prior reports,^29,41,57^ with fastest production at pH 3 and progressively decreasing formation rates with increasing pH. These results confirm a clear shift in radical generation pathways with pH, with less selective, stronger oxidants prevailing at lower pHs, prompting a question of the role and efficacy of the more selective oxidants that occur at higher pHs and longer wavelengths.

**Figure 1:**
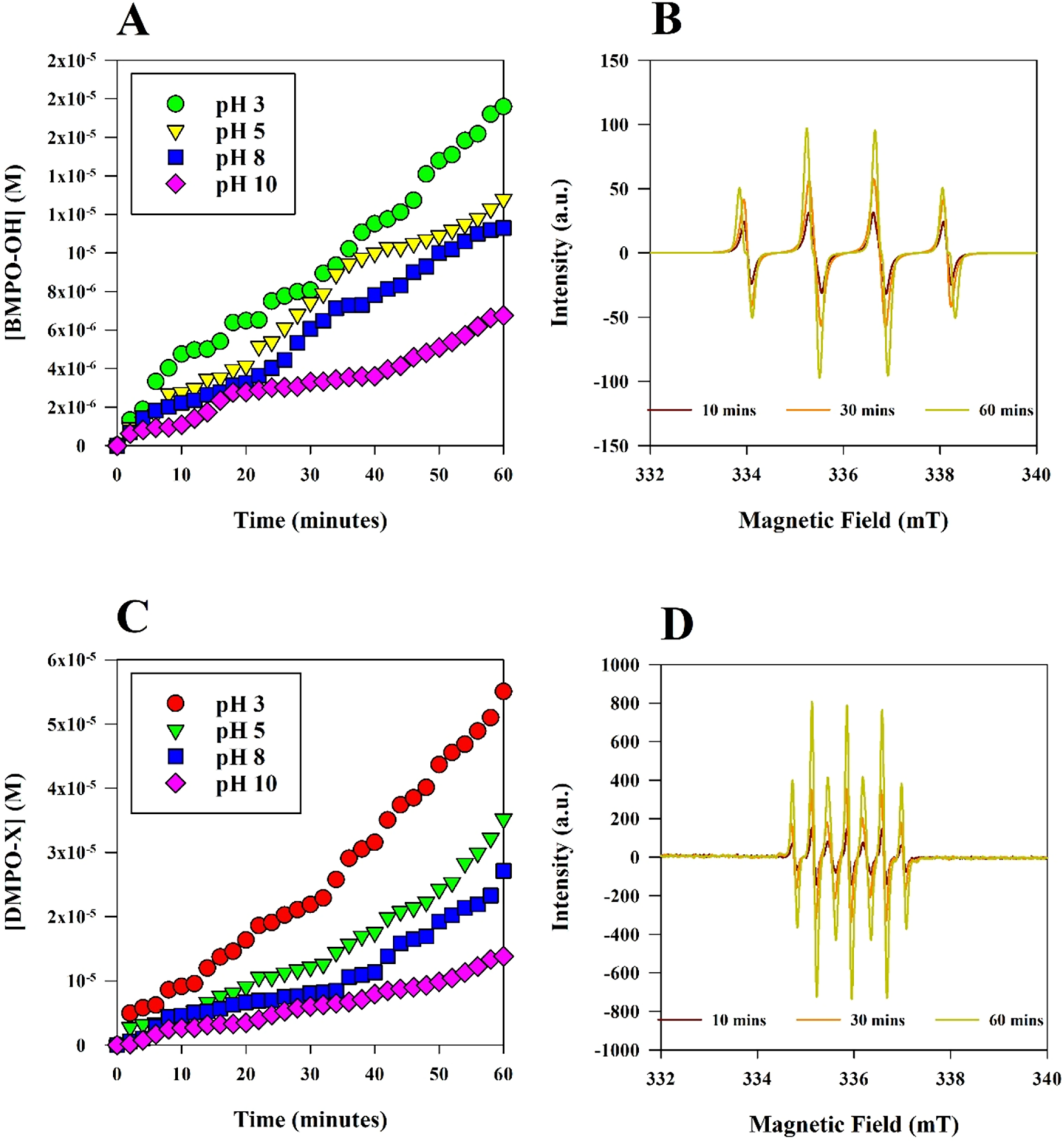
(A) BMPO–OH radical adduct formation over 60 min of UV_305_/Cl at pHs 3, 5, 8, and 10, and (B) the corresponding simulated EPR spectra with the following parameters: initial chlorine concentration = 10 mg/L-Cl_2_; initial BMPO concentration = 300 mM; hyperfine splitting constants of BMPO–OH, *a*N = 13.4454 G, *a*β-H = 14.5894 G, *a*γ-H = 0.709 G; and *g*-factor = 2.00651. (C) DMPO-X radical adduct formation over 60 min of UV_305_/Cl at pHs 3, 5, 8, and 10, and (D) the corresponding simulated EPR spectra with the following parameters: initial chlorine concentration = 10 mg/L-Cl_2_; and initial DMPO concentration = 300 mM.

### The Role of O_3_ in CBZ Degradation

To quantify O_3_ production in the UV_305_/Cl system, experiments were conducted with and without dissolved oxygen (DO). The reaction vessel was modified for this experiment to allow the purging of solution and headspace by N_2_ before and during reactions to remove and preclude DO. O­(^3^P), is produced during the photolysis of OCl^–^,^41^ which undergoes wavelength-dependent cleavage pathways that generate O^–•^, O­(^1^D), and O­(^3^P); the latter then reacts with dissolved O_2_ to form O_3_.^41^ Since O_3_ is formed via reactions between O_2_ and O­(^3^P), removing DO precludes O_3_ formation. Experiments with DO present showed that O_3_ concentrations varied across different pH values with the highest values observed under slightly basic conditions, as shown in Table 1. This observation contrasts with the conventional dynamics of ozonation systems, where increasing pH promotes O_3_ decay via reactions with OH^–^.^58^ In such systems, O_3_ is more stable at lower pHs, and the reactions with hydroxide generates H_2_O_2_, ultimately leading to ^•^OH formation, under basic conditions.^58,59^ In the UV/Cl system, O_3_ is generated through a completely different pathway, and the speciation of free chlorine explains the pH dependence observed here. O_3_ accumulation peaked at pH 8 under UV_305_, consistent with other reports,^41,60–62^ yielding a concentration of 1.97 μM O_3_ when 5 mg-Cl_2_/L was irradiated. According to Bader and Hoigné,^63^ the indigo method employed by the Hach AccuVac ampules enables a rapid and reliable determination of O_3_ in water, provided that the sample is analyzed immediately and is free from other oxidizing or reducing agents. However, interferences may arise from strong oxidants and reducing species, which can cause overestimates or underestimates, respectively the effective ozone concentration available to react with the indigo dye. Here, the N_2_ purged controls, showing no change in indigo concentration, indicate that the O_3_ measurements are not overestimated by the presence of free chlorine or its direct photolysis products. Of the numerous reactions reported for chlorine photolysis, the only pathway involving O_2_ as a reactant is the formation of O_3_ by reaction with O­(^3^P).^29,64,65^

**Table 1:** Concentrations of O_3_ in DI Water during UV/Cl Treatment at 254 and 305 nm with and without Dissolved Oxygen Using 5 mg-Cl_2_/L at Varying pH Values

	UV_254_		UV_305_	
pH	O_3_ concentration with oxygen (μM)	O_3_ concentration without oxygen (μM)	O_3_ concentration with oxygen (μM)	O_3_ concentration without oxygen (μM)
5	0.14	0.0	0.42	0.0
7	0.32	0.0	1.21	0.0
8	0.64	0.0	1.97	0.0
9	0.11	0.0	0.37	0.0

CBZ removal experiments were conducted at pH 5 and pH 8, with and without DO (Figure 2). Linear regressions and *k*_obs_ values for these data are shown in Figure S2. At pH 5 with DO, the *k*_obs_ for CBZ degradation was 5.67 ± 0.006 × 10^–3^ s^–1^. In the absence of DO at pH 5, CBZ removal was significantly slowed, such that *k*_obs_ = 2.28 ± 0.02 × 10^–3^ s^–1^. At pH 8, the *k*_obs_ were found to be 1.23 × 10^–2^ s^–1^ and 2.0 × 10^–4^ s^–1^ with and without DO, respectively, revealing that the presence of O_2_ was especially important in the basic condition. At low pHs, the dominance of HOCl leads to the generation of ^•^OH and Cl^•^/Cl_2_^•–^ as primary radicals in the UV_305_/Cl system. In the basic region, O dominates and generates several different radical species during photolysis: O­(^1^D), O­(^3^P), Cl_2_^•–^, ClO^•^, and ^•^O^–^ (the conjugate base of ^•^OH).^41,57,61,62,66–68^ Under both pH conditions, the absence of DO suppressed CBZ degradation by precluding O_3_ production.

**Figure 2:**
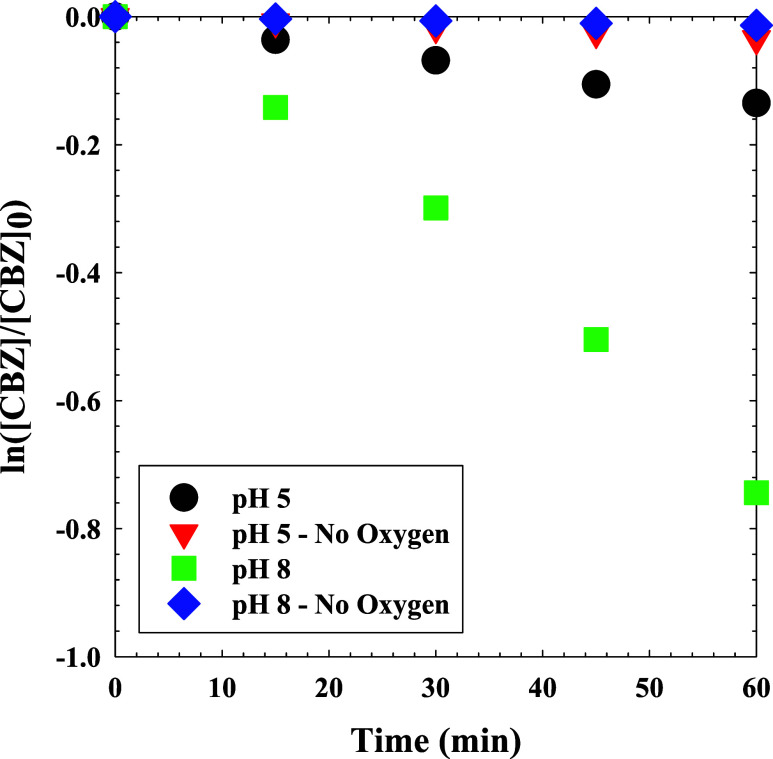
Degradation kinetics of CBZ (25 μM) during UV_305_/Cl process using 5 mg-Cl_2_/L at pH 8 and pH 5 in the presence and absence of oxygen.

The increased degradation of CBZ at pH 8 compared to pH 5 matched well with the increased O_3_ accumulation in the system. We estimate that more than 60% of the reaction at pH 8 was caused by O_3_, according to the approach taken by Hua et al. in 2019.^62^ It is possible for O_3_ to then photodegrade to form singlet oxygen (^1^O_2_) and hydroxyl radicals, but these are not expected to be major contributors, since O_3_ absorbs weakly at 300 nm.^40,41,62,69,70^ With an oxidation potential of 2.07 V, O_3_ can react with a large number of pollutants. In addition to O_3_ formation in the system, the increased degradation of CBZ at high pH may be attributed in part to reactions with ClO•, which is a selective oxidant with a redox potential between 1.5 and 1.8 V.^1,57,71,72^ At higher pHs, the rate constant for the generation of ClO^•^ is about 3 times higher than at lower pH values.^1,57,71^ Other studies have reported the concentration of ClO^•^ to be 3 to 4 orders of magnitude higher than ^•^OH, Cl^•^, Cl_2_^•^, playing a role in the degradation of other micropollutants in UV_305_/Cl systems at high pH.^1,22,57,73–77^ ClO^•^ is likely the species responsible for the destruction of CBZ at pH 8 in the absence of oxygen and O_3_.

### Effect of Solution pH on CBZ Degradation

The impacts of solution pH on the degradation of CBZ by chlorine or UV_305_/Cl were examined at pHs 3, 5, 8, and 9. Dark controls to quantify CBZ destruction by free chlorine alone were also performed. Linear regressions and *k*_obs_ values for these data are presented in Figure S3. Generally, free chlorine did not degrade much CBZ except for the pH 3 case, which exhibited a *k*_obs_ of 5.40 ± 0.2 × 10^–3^ s^–1^, as shown in Figure 3a. At pH 3 Cl_2_(g) forms via the reaction of HOCl and Cl^–^, and the further dissociation of HOCl yields a minor species of Cl_2_O. The Cl_2_ and Cl_2_O were likely responsible for the degradation of CBZ at pH 3.^78^ Kinetic modeling and experimental data from Yang et al. indicate that under mildly acidic conditions (pH 5), Cl_2_ and Cl_2_O contributed 65.2% and 29.3% to CBZ degradation, respectively, while HOCl accounted for only 5.5%. Their work revealed that CBZ removal by free chlorine under acidic conditions was primarily driven by Cl_2_ and Cl_2_O, rather than ^•^OH.^78^ Soufan et al. in 2013 also reported a similar observation for CBZ degradation by free chlorine at neutral to acidic pHs, with an initial chlorine and CBZ concentrations of 10 mg-Cl_2_/L and 100 μM, respectively.^79^ At pH values above 8, on the other hand, free chlorine had a negligible impact on CBZ degradation. The UV_305_/Cl process, however, removed CBZ effectively, especially at pHs 3 and 8, as depicted in Figure 4b. Over 60 min, removals of 80% and 74% were achieved for CBZ under UV_305_/Cl at pHs 3 and 8, respectively. These pH-dependent degradation behaviors directly align with the EPR analyses of the UV/Cl system (Figure 1), which demonstrated that radical generation efficiency is strongly governed by solution pH. Specifically, EPR spectra revealed that BMPO–OH and DMPO–X adduct formation rates peaked at pH 3 and progressively decreased with increasing pH, confirming that both ^•^OH and Cl^•^/Cl_2_^•–^ production is favored under acidic conditions.

**Figure 3:**
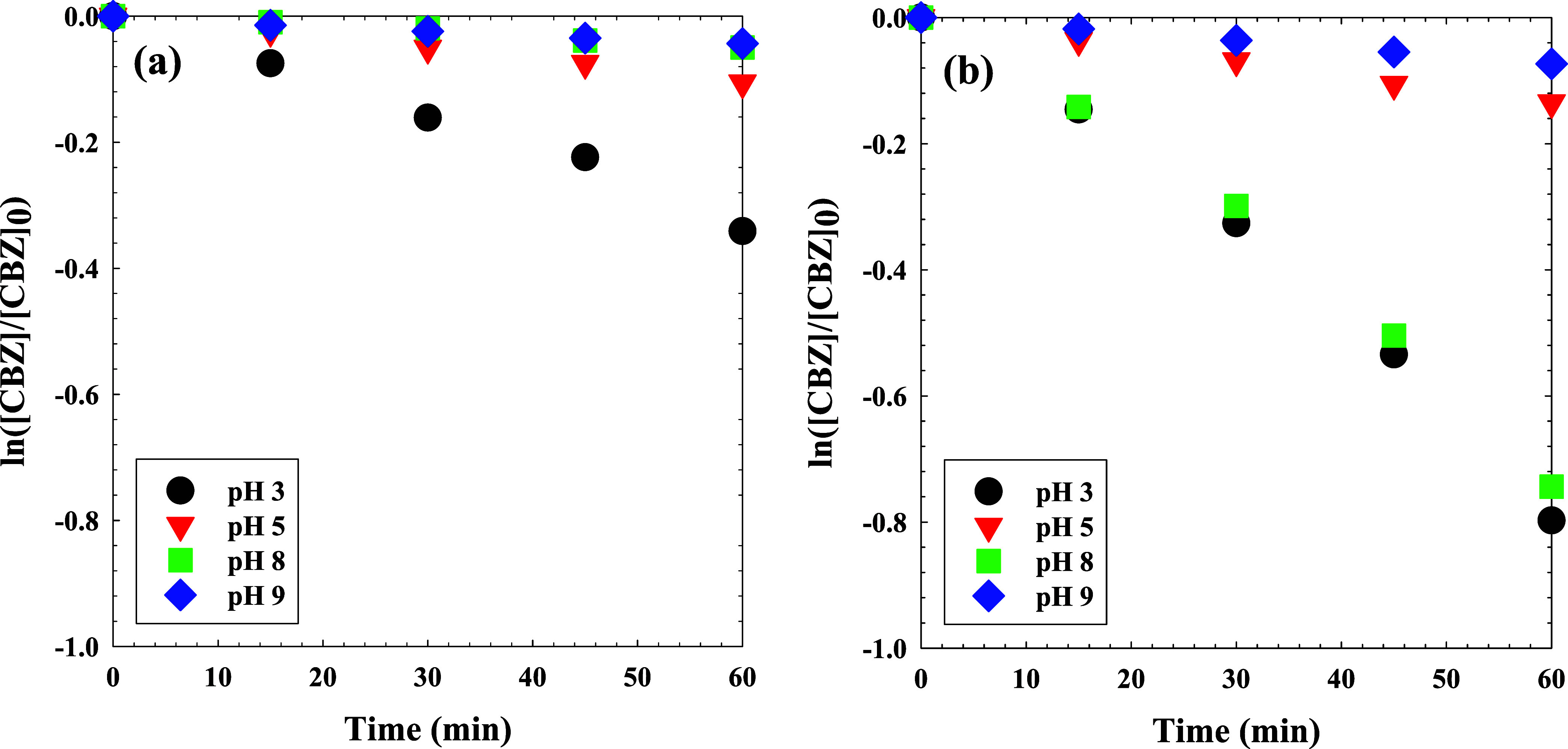
Degradation kinetics of CBZ (25 μM) using 5 mg-Cl_2_/L at varying pH values under (a) dark conditions and (b) UV_305_ irradiation.

**Figure 4:**
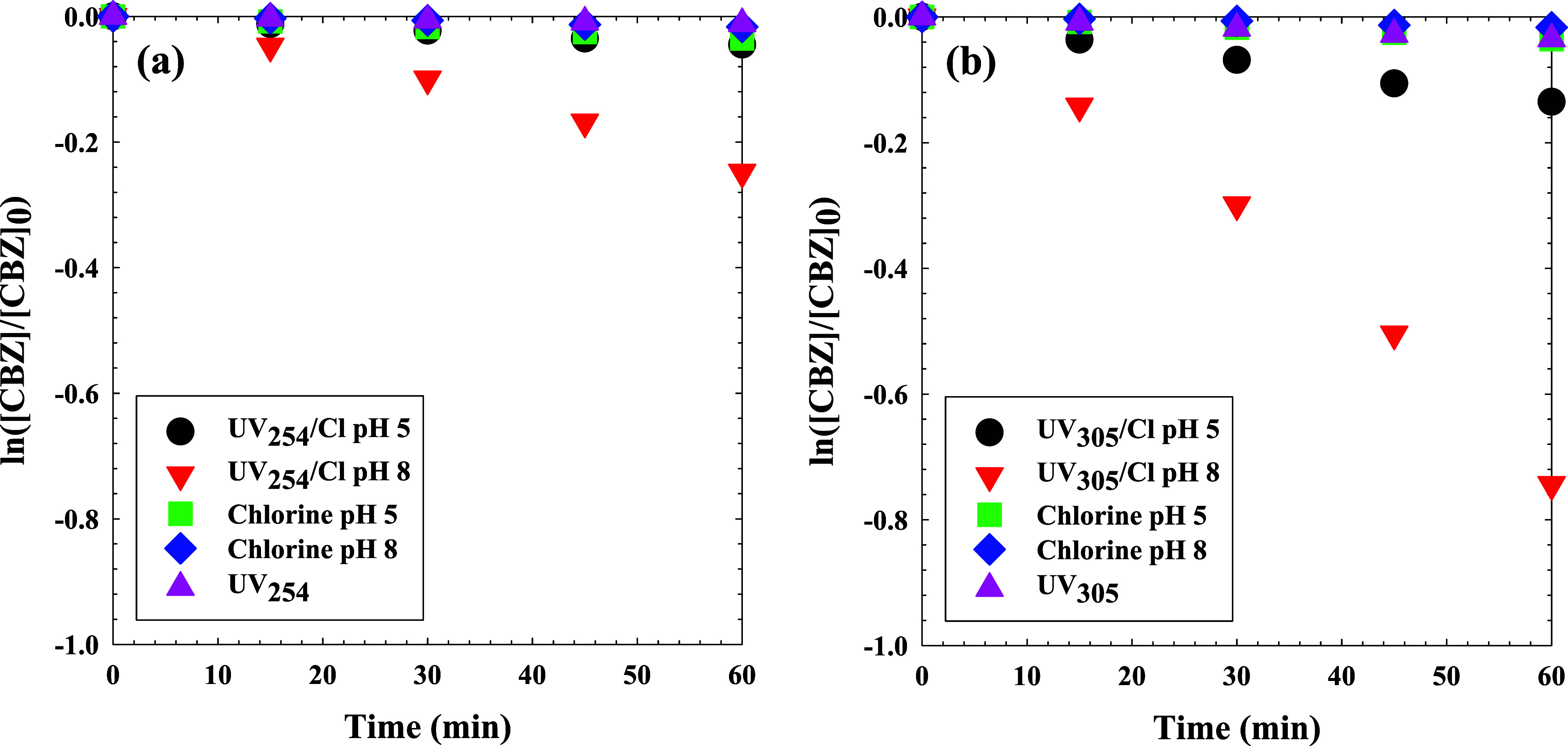
Degradation kinetics of 25 μM CBZ by 5 mg-Cl_2_/L with (a) UV_254_/Cl, free chlorine only (dark), UV_254_ irradiation only, or (b) UV_305_/Cl, free chlorine only (dark), UV_305_ irradiation only.

Aside from the O_3_ effects discussed, several other factors may also contribute to the pH dependencies observed in the UV_305_/Cl system. First, the molar absorptivity of OCl^–^ at 305 nm is higher than that of HOCl, providing improved UV_305_ absorbance at basic pHs.^9,41,62,80^ Therefore, at high pHs, there was an enhancement in chlorine photolysis that generated radical species capable of degrading CBZ. At acidic pHs, the photolysis of HOCl yields ^•^OH and Cl^•^.^20,41^ This interpretation is further supported by the EPR results, which confirmed that the yields of BMPO–OH and DMPO–X adducts were highest at low pH, mirroring the dominant contribution of ^•^OH and Cl^•^/Cl_2_^•–^ radicals to CBZ degradation in the same range. Basic conditions promote OCl^–^ photolysis which was already described: generating ^•^O^–^, O­(^1^D), O­(^3^P), Cl_2_^•–^, ClO^•^, and ultimately O_3_ (Table 1).^41,57,61,62,66–68^ Recent reports demonstrated effective O_3_-driven degradation of CBZ at high pHs and UV wavelengths higher than 300 nm in UV/Cl systems.^9,81^ In the present study, a higher *k*_obs_ of 1.16 ± 0.5 × 10^–2^ s^–1^ at pH 8 is likely caused by the O_3_ production. At pH 9, however, a significant decrease in O_3_ (0.37 μM) and a corresponding lower *k*_obs_ of 1.24 ± 0.5 × 10^–2^ s^–1^ was observed. A recent study by Wang et al., in 2017 reported that there is more radical quenching of ROS and RCS in the UV/Cl system during CBZ degradation at pH values greater than 8.5.^9,81^ The rates of ^•^OH and RCS quenching by aqueous free chlorine is higher in basic solutions (OCl^–^) compared to acidic solutions (HOCl).^9^ Their observation is consistent with the present study where lower *k*_obs_ was also observed at pH 9 due to radical quenching of ^•^OH and RCS by HCO_3_^–^ in the UV/Cl system. This phenomenon could account for a lower concentration of O_3_ generated at pH 9 and a resulting lower *k*_obs_ of CBZ. The degradation of CBZ at pH 3 (Figure 3a) was similar to the pH 8 case (Figure 3b). The reason for the higher rate of CBZ degradation could be attributed to a combination of reactions with Cl_2_(g) and the predominance of ^•^OH during the photolysis of HOCl at pH 3.^41^ Reactions with colloidal DOM showed that the UV_305_/Cl process was strongly inhibited at pH 5 compared to pH 8 (Figure S5), providing further support for the notion that hypochlorite photolysis may be the more efficient pathway for UV/Cl. The *E*_EO_ values for CBZ removal were determined at pH 3 and pH 8 using the Bolton method.^44^ The recorded *E*_EO_ at pH 3 was 0.0563 kWh/order·m^3^, which is a bit lower than the 0.0604 kWh/order·m^3^ observed at pH 8. This difference demonstrates that the UV/Cl system achieved higher energy efficiency at pH 3. However, adjusting the pH to such a low level is impractical for cost and safety reasons (Cl_2_(*g*) off-gassing) in large-scale water treatment applications.

### Wavelength Dependence of CBZ Degradation

The effects of irradiation sources on UV/Cl-driven CBZ degradation were explored by conducting experiments under UV_254_, UV_305_, or chlorine only (dark), each at pH 5 and pH 8. These data are plotted in Figures 4 and S4, and Figure S5 present the corresponding linear regression analyses and fluence-based and time-based *k*_obs_ values. Two wavelengths, UV_254_ (UVC) and UV_305_ (UVB) were selected to examine possible advantages of wavelength selection afforded by UV LEDs. Standard low-pressure mercury lamps emit at 253.5 nm, near a moderate absorption peak of HOCl. Conversely, longer-wavelength LEDs provide emissions that can match the much stronger absorption peak of OCl^–^, yielding a significantly higher molar absorption coefficient than HOCl at 254 nm.^80^ To facilitate a meaningful comparison between UV wavelengths, degradation kinetics were also assessed as a function of UV fluence (J/cm^2^) in Figure S5 of the SI. The UV_305_/Cl process exhibited a faster degradation rate than UV_254_/Cl, especially at pH 8, where *k*_obs_ increased from −3.31 ± 0.01 × 10^–1^ cm^2^/J for UV_254_/Cl to −4.23 ± 0.02 × 10^–1^ cm^2^/J for UV_305_/Cl. In contrast, direct photolysis and chlorine-only reactions showed significantly lower fluence-based rate constants. Without chlorine, UV_254_ and UV_305_ were similarly ineffective at degrading CBZ. At pH 8 and an initial chlorine concentration of 5 mg-Cl_2_/L, about 30% and 98% of CBZ were eliminated after 60 min by UV_254_/Cl and UV_305_/Cl, respectively. Degradation of CBZ via chlorination in the dark was slow at these pHs. The UV_305_ drove faster CBZ removal at pH 8, with *k*_obs_ = 1.12 ± 0.5 × 10^–2^ s^–1^, compared to UV_254_ at either pH 8 (*k*_obs_ = 3.87 ± 0.2 × 10^–3^ s^–1^) or at pH 5 (*k*_obs_ = 7.62 ± 0.008 × 10^–4^ s^–1^) as shown in Figure 4a. Notably, the UV_305_/Cl *k*_obs_ was higher, measuring 1.12 ± 0.5 × 10^–2^ s^–1^ at pH 8, than the UV_254_/Cl system at pH 8, which had a *k*_obs_ of 4.10 × 10^–3^ s^–1^, and these trends held true on a fluence basis as well. The pronounced improvement in CBZ removal at pH 8 by UV_305_ over UV_254_ is likely a function of additional O_3_ formed as shown in Table 1, given that OCl^–^ has a higher molar absorptivity at 305 nm than HOCl. Even though the difference in molar absorptivities is offset by a higher quantum yield for HOCl (ϕ_310_ = 1.19 mol/Einstein) compared to OCl^–^ (ϕ_310_ = 0.39 mol/Einstein),^9,82^ a higher portion of free chlorine is converted to O_3_ at pH 8 under UV_305_, yielding a more efficient process. Yin et al. in 2018 also reported higher fluence-based rate constants at higher pH and longer wavelength compared to those at lower pHs in the UVC range.^83^ Bulman et al. reported the formation of O_3_ at 254 nm at higher pHs and attributed it to the higher molar absorptivity of OCl^–^ at higher pH.^41^ This finding was consistent with the observation that meaningful degradation (*k*_obs_ = 3.87 ± 0.2 × 10^–3^ s^–1^) of CBZ was observed at UV/Cl_254_ at pH 8 (Figure 4a).

### ROS/RCS Quenching Experiments

Quenching experiments were conducted to identify the key ROS/RCS responsible for CBZ degradation in the UV/Cl system, as shown in Figure 5A and summarized in Table S4 of the SI. Quenchers were selected based on their reactivities with the species of interest, as depicted in Figure 5B and tabulated in Table S5 of the SI, which plots reported rate constants (M^–1^ s^–1^) of key ROS and RCS with CBZ and each quencher.^41,84–100^ CBZ degraded fastest without quenchers (*k*_obs_ = 1.933 ± 0.003 × 10^–4^ s^–1^), as expected, reflecting the combined oxidative activity of all reactive species produced during chlorine photolysis. The addition of TBA inhibited the rate of CBZ removal by nearly half (*k*_obs_ = 1.196 ± 0.001 × 10^–4^ s^–1^), attributable to the significant quenching of ^1^O_2_ and moderate competition for ^•^OH by TBA. NBZ further inhibited CBZ degradation, yielding a *k*_obs_ of 1.033 ± 0.0009 × 10^–4^ s^–1^, acting as a stronger competitor for ^•^OH (10^8^ M^–1^ s^–1^) than TBA. SBZ caused still stronger inhibition (*k*_obs_ = 9.368 ± 0.0009 × 10^–5^ s^–1^). SBZ effectively quenches ^•^OH, ^1^O_2_, and Cl^•^ at near diffusion-controlled rates (∼10^9^ M^–1^ s^–1^) and it competes with a similar reactivity toward Cl_2_^•–^, but SBZ is only mildly competitive with ClO^•^ (∼10^5^–10^6^ M^–1^ s^–1^) and does not quench O_3_ effectively. SAZ induced the greatest inhibition, reducing the *k*_obs_ to 7.564 ± 0.002 × 10^–5^ s^–1^. Although SAZ is often used as a selective quencher for ^1^O_2_ (∼10^9^ M^–1^ s^–1^), it also reacts rapidly with ^•^OH (>10^9^ M^–1^ s^–1^) and moderately so with O_3_ (∼10^5^ M^–1^ s^–1^). Of all quenchers, SAZ is the only one to out-compete CBZ in reactivity with O_3_, and it caused the most inhibition. Clearly, multiple oxidants contribute to CBZ removal, but O_3_ stands out as a key ingredient. The resulting rate constants and reactive species concentrations for UV/Cl_305_ at pH 8 were derived according to Text S2 and summarized in Tables S4, S5, and S6 of the SI. Notably, the addition of SAZ caused a ∼61% reduction in CBZ removal rate whereas TBA only reduced the rate by ∼38%; both compounds react competitively with ^•^OH and ^1^O_2_, but SAZ also competes for O_3_. SBZ, which is competitive—if not efficient—against all reactive species except O_3_, reduced the *k*_obs_ by ∼51%, suggesting that O_3_ contributes to about half of the CBZ removal under these conditions.

**Figure 5:**
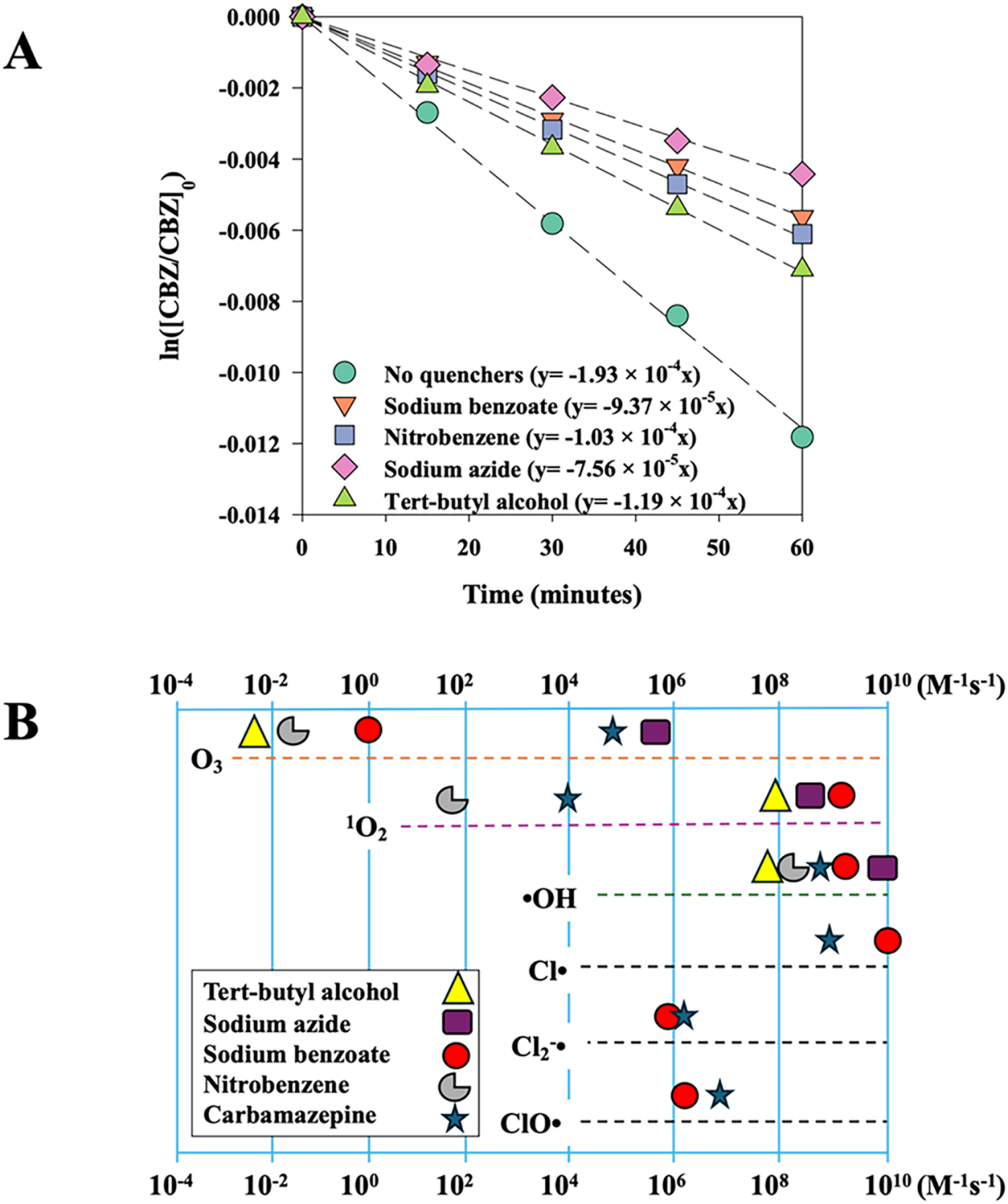
(A) Degradation of 25 μM CBZ by 5 mg-Cl_2_/L with UV_305_/Cl at pH 8 in the absence or presence of 1 mM of quencher molecules: tert-butyl alcohol, sodium azide, sodium benzoate, or nitrobenzene; and (B) the comparison of bimolecular rate constants (M^–1^ s^–1^) of selected oxidants with CBZ and the quenching compounds.^41,84–99^

### DOM Interference in CBZ Degradation

The unprecedented control over ROS/RCS in the UV/Cl system afforded by the pH- and wavelength-dependent photochemistry provides a novel approach to overcoming DOM interference in AOP operation. Specifically, for a given combination of pH and DOM type, an irradiation wavelength can be selected for optimal performance. A series of experiments was performed with different DOM isolates (colloidal, HPO, TPI, and HA) at pH 3 and pH 8 under UV_305_ to delineate the utility of ROS/RCS selection on inhibition avoidance (Figure 6). UV_305_ irradiance and the initial chlorine concentration (5 mg Cl_2_/L) were kept constant for these experiments, and the available chlorine was observed to be maintained above 3 mg Cl_2_/L over the 60 min experimental time frame, as shown in Figure S10 of the SI. A control experiment with no DOM was also conducted. Each DOM isolate inhibited CBZ destruction to some extent, and the degree of inhibition varied with DOM type and solution pH. Absent DOM, the UV_305_/Cl process achieved significant CBZ degradation with *k*_obs_ of 1.24 ± 0.5 × 10^–2^ s^–1^ for the 60 min treatment period. All DOM isolates inhibited the CBZ removal significantly at pH 3, with HA retarding the process the most by a factor of 8. At this pH, the colloidal, HPO, and TPI fractions exerted similar inhibitory effects, yielding similar *k*_obs_ values about four times lower than the No DOM case shown in Figure 6a (regression analyses shown in Figures S6 and S7). The presence of 1 mg-C/L colloids reduced the degradation rate of CBZ by up to 95% at pH 3 and 80% at pH 8. Given that the dominant radical species expected from UV_305_/Cl at pH 3 is the nonselective ^•^OH,^20,41^ the drastic reduction in CBZ removal efficiency is unsurprising. Cl^•^ and Cl_2_^•–^ are also expected in the system at pH 3 and these are more selective than ^•^OH, likely contributing to the CBZ removal that was observed.

**Figure 6:**
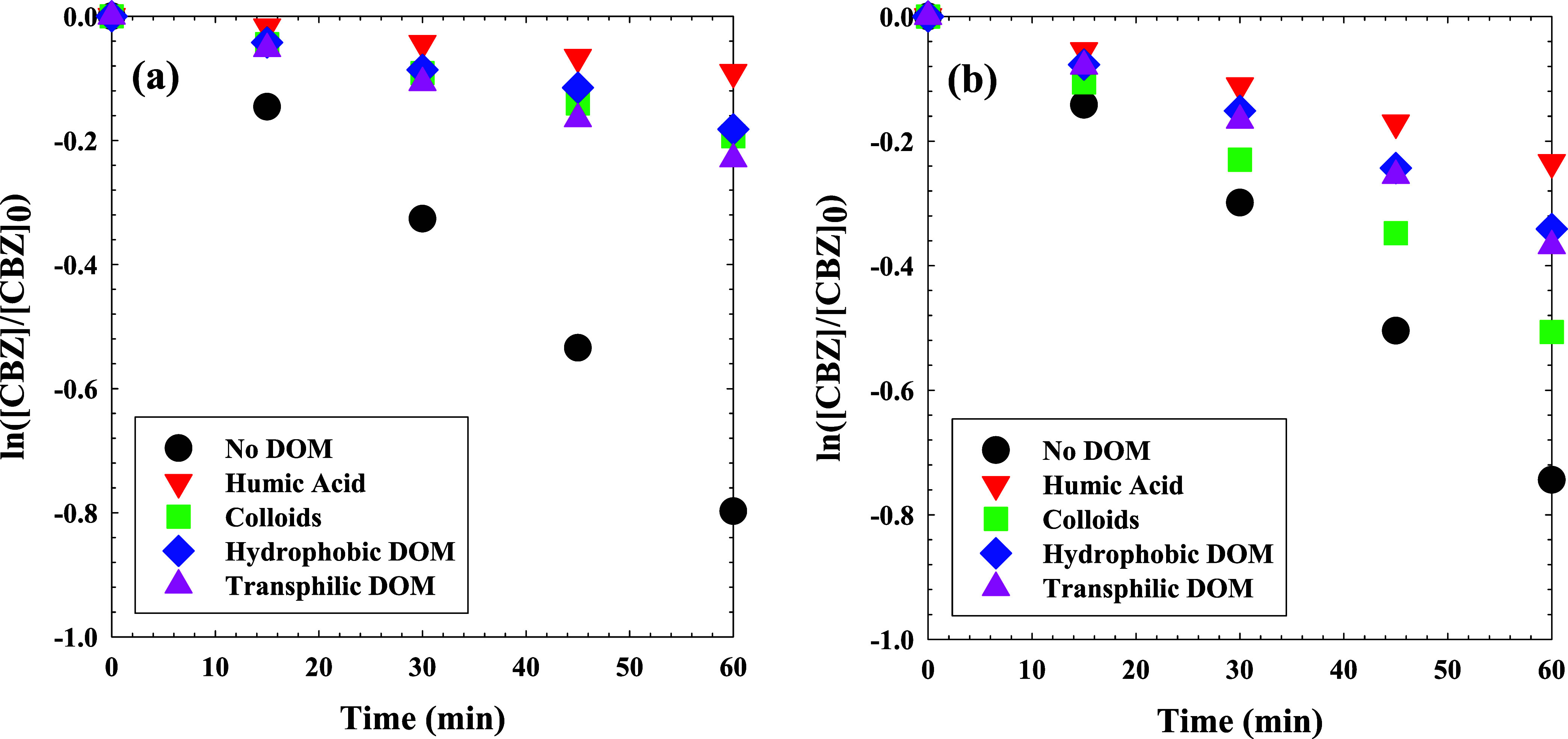
Effect of 1 mg-C/L of different DOM isolates on the UV_305_/Cl AOP using 5 mg-Cl_2_/L for the degradation of CBZ (25 μM) at (a) pH 3 and (b) pH 8.

The interference of DOM on the UV_305_/Cl system at pH 8 yielded a much different outcome. Unlike the pH 3 case, the inhibition of CBZ removal was not as strong for the DOM isolates, as shown in Figure 6b. The process at pH 8 achieved a *k*_obs_ of 1.16 ± 0.5 × 10^–2^ s^–1^ with no DOM present, comparable to 1.24 ± 0.5 × 10^–2^ s^–1^ in the pH 3 case. HA exerted the strongest inhibition, followed by HPO and TPI which were similar in effect, then colloids, which only reduced the *k*_obs_ to 8.08 ± 0.2 × 10^–3^ s^–1^. The process in the presence of HA reduced the *k*_obs_ by 68.3% at pH 8, compared to 88.6% at pH 3. Significant amounts of O_3_ were generated in the system at pH 8 (Table 1), providing a more selective oxidant for CBZ destruction. Accordingly, the colloids were apparently least reactive with O_3_, with a CBZ removal *k*_obs_ of 8.08 ± 0.2 × 10^–3^ s^–1^ under these conditions. Fu et al. reported that O_3_ reacts with low-molecular-weight components of DOM isolates and increases their fluorescence intensity.^101^ By definition, colloids have molecular weight greater than 100 kDa.^28^ Among the DOM tests, the fastest degradation of CBZ was observed with colloids, plausibly linked to the lower affinity of O_3_ to react with the colloidal fraction due to their size and proteinic nature.^28^ These proteinaceous colloids are relatively less enriched in aromatic and polyaromatic moieties than HPO and TPI fractions, which are instead mainly composed of humic- and fulvic-like structures with more aromaticity and hydrophobicity. Because O_3_ reacts more readily with electron-rich aromatics than with aliphatic or peptide backbones,^101^ the lower apparent O_3_ inhibition by colloidal DOM at pH 8 may indicate an inherently lower O_3_ demand rather than merely restricted ozone access to reactive moieties or molecular size. The observed rate constants of HPO and TPI, on the other hand, were almost the same (5.49 ± 0.1 × 10^–3^ s^–1^ and 5.89 ± 0.1 × 10^–3^ s^–1^ respectively). HPO and TPI fractions, studied in depth elsewhere,^28^ were shown to contain similar amounts of diacids, and polyaromatic hydrocarbons and have similar molecular weights at around 1 kDa,^28^ which could explain the similar inhibition outcomes. The higher inhibition observed with HPO and TPI potentially stemmed from their characteristic protein-like, fulvic-like, and humic-like substances which allow them to react with O_3_ thereby inhibiting CBZ oxidation.^28,101^

### DOM Reactivity and Transformation

To better understand the extent and nature of DOM reactivity in the UV_305_/Cl system, the HPO and colloidal fractions were characterized via 3DEEM fluorescence spectroscopy before and after treatment at pH 8. HPO and TPI were shown to be similar in profile in the results above and in our previous work,^28^ so HPO is used as a representative for both here. The total fluorescence volumes were quantified within defined regions; these data are presented in Figure 7. The fluorescence compositions of HPO and colloids were quite distinct, with fulvic-like (zone III) and humic-like (zone V) fluorophores comprising more than 80% of the HPO fluorescence. These fluorophores are associated with aromatic and conjugated structures that are known to react strongly with O_3_ and other electrophilic oxidants, especially under basic conditions where O_3_ dominates. The high proportion of fluorescing compounds in zones III and V in HPO possibly indicates that the HPO may inhibit more O_3_ than colloids in this condition. Recent studies on photo-Fenton treatment and photocatalysis showed that both humic-like and fulvic-like fluorophores impact micropollutant removal.^102–104^ Fulvic-like and humic-like substances inhibit the photodegradation of organic micropollutants by scavenging ROS that react with electron-rich moieties of the DOM.^102,105^ Humic-like substances have high aromatic carbon content, lignin, and polyphenols, which exhibit stronger reactivity with ROS, thereby quenching them before they can degrade pollutants.^102,105^ Fulvic-like substances, on the other hand, have higher polarity and abundant carboxyl groups; these groups also scavenge ROS, albeit to a lesser extent, given their lower aromaticity.^28,102,105^ In contrast, the protein- and SMP-rich colloidal fraction has fewer aromatic reactive sites, which likely limits its direct O_3_ reactivity and decreases its inhibitory effect under UV_305_/Cl conditions. Thus, the high proportion of fluorescing compounds in zones III and V in HPO suggests that the HPO may be more reactive with ROS than colloids in degrading micropollutants. Hence, HPO could be expected to exhibit more inhibition than the colloids.

**Figure 7:**
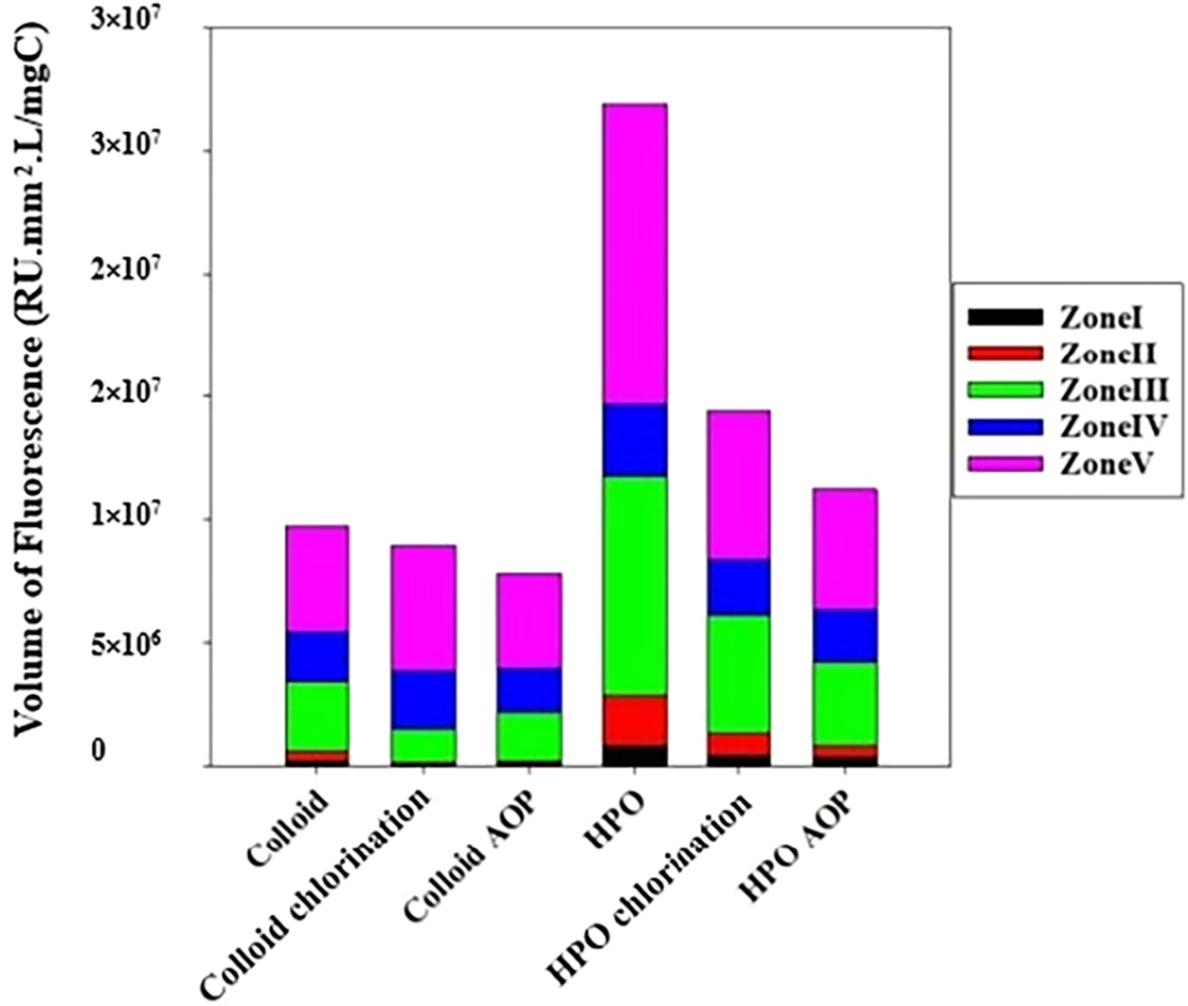
Fluorescence (a) percentage and (b) volume determined for the colloidal, and HPO fractions before and after exposure to UV_305_/Cl, at pH 8, using 5 mg-Cl_2_/L. Solutions were prepared with DOM concentrations of 1 mg-C/L. Regions are categorized as (I) aromatic protein-like, type I; (II) aromatic protein-like, type II; (III) fulvic acid-like; (IV) SMP-like; and (V) humic acid-like fluorophores.^47^

The HPO and colloidal organics were compared for fluorophore content on a carbon basis before and after treatment by free chlorine or UV/Cl (Figure 7). Normalized by TOC, HPO contained more fluorophores than the colloidal fraction. Fluorescence signals from both isolates were reduced by chlorination and even further by UV_305_/Cl. Free chlorine oxidized the protein-like types I and II (zones I and II) and fulvic-like (zone III) in colloids. New moieties were formed during chlorination that changed the fluorescence signal, increasing emissions in zones IV and V. UV_305_/Cl was reactive with colloids, changing the fluorescence volume in each zone by destroying corresponding fluorophores. In the case of HPO, chlorination and UV_305_/Cl both curtailed the fluorescence in each zone, with UV_305_/Cl causing twice the reduction of fluorescence than that of chlorination. Given that O_3_ is predominant at basic conditions, it likely played a crucial role in oxidizing fluorophores in zones I, II, III, and V of HPO in the UV_305_/Cl system.^101,106^ Nevertheless, the SMP-like fluorophores in zone IV showed minimal reactivity, with almost no reduction under chlorination or UV_305_/Cl, suggesting little reactivity of fluorophores in that region with O_3_. This behavior reflects the inherently lower direct reactivity of SMP-associated, proteinaceous, or aliphatic moieties with O_3_, which lack electron-rich aromatic structures. As a result, the limited fluorescence loss in zone IV indicates the relative chemical stability of these DOM components during UV_305_/Cl treatment.

### CBZ Degradation: Microtoxicity Assay and Byproduct Assessment

The acute toxicity of CBZ and its resulting transformation products after UV_305_/Cl are shown in Figure 8. Residual free chlorine was quenched with sodium thiosulfate immediately after the UV/Cl process, prior to toxicity assessment. Panel A shows the luminescence inhibition of *V. fischeri* following 5 and 15 min of exposure to the UV/Cl-treated CBZ solutions. At time zero, the solution caused minimal luminescence inhibition (∼8%). After 60 min of UV/Cl treatment, luminescence inhibition increased to ∼14%. The CBZ degradation byproducts were slightly more toxic compared to the parent compound. The transformation products (labeled here as P1–P8, as detailed in Table S7 of the Supporting Information) were identified based on their molecular structures and mass fragmentation patterns. Figure 8B shows the evolution of these CBZ transformation products over an hour treatment period. Among the identified products, CBZ epoxide (P5) was the dominant species, with its concentration increasing steadily to ∼0.71 μM at 60 min. The accumulation of P5 likely contributed to the elevated acute toxicity observed. At the same time, the chlorinated intermediates (P6 and P7) warrant attention, because halogenation can increase hydrophobicity and biological potency even at low levels,^107^ and these species may disproportionately contribute to the observed inhibition. CBZ diols (P1), the chloroalcohol derivative (P4), and the monochlorinated species (P6) were detected at lower concentrations (0.0741 μM, 0.0247 μM, 0.0297 μM, respectively), whereas the other intermediates (P2, P3, P6, P7, and P8) occurred only at trace levels (0.000486 μM–0.00797 μM). The observed toxicity thus falls within the range reported for comparable AOPs, and is generally seen as indicating relatively low acute toxicity. These findings are consistent with earlier findings for other AOPs where transient accumulation of CBZ intermediates caused similar toxicity responses.^108–110^ The formation of chlorinated byproducts (P6 and P7), confirmed by distinctive chlorine isotopic patterns, suggests that halogenated intermediates can be generated and then persist during CBZ degradation in the UV/Cl system.

**Figure 8:**
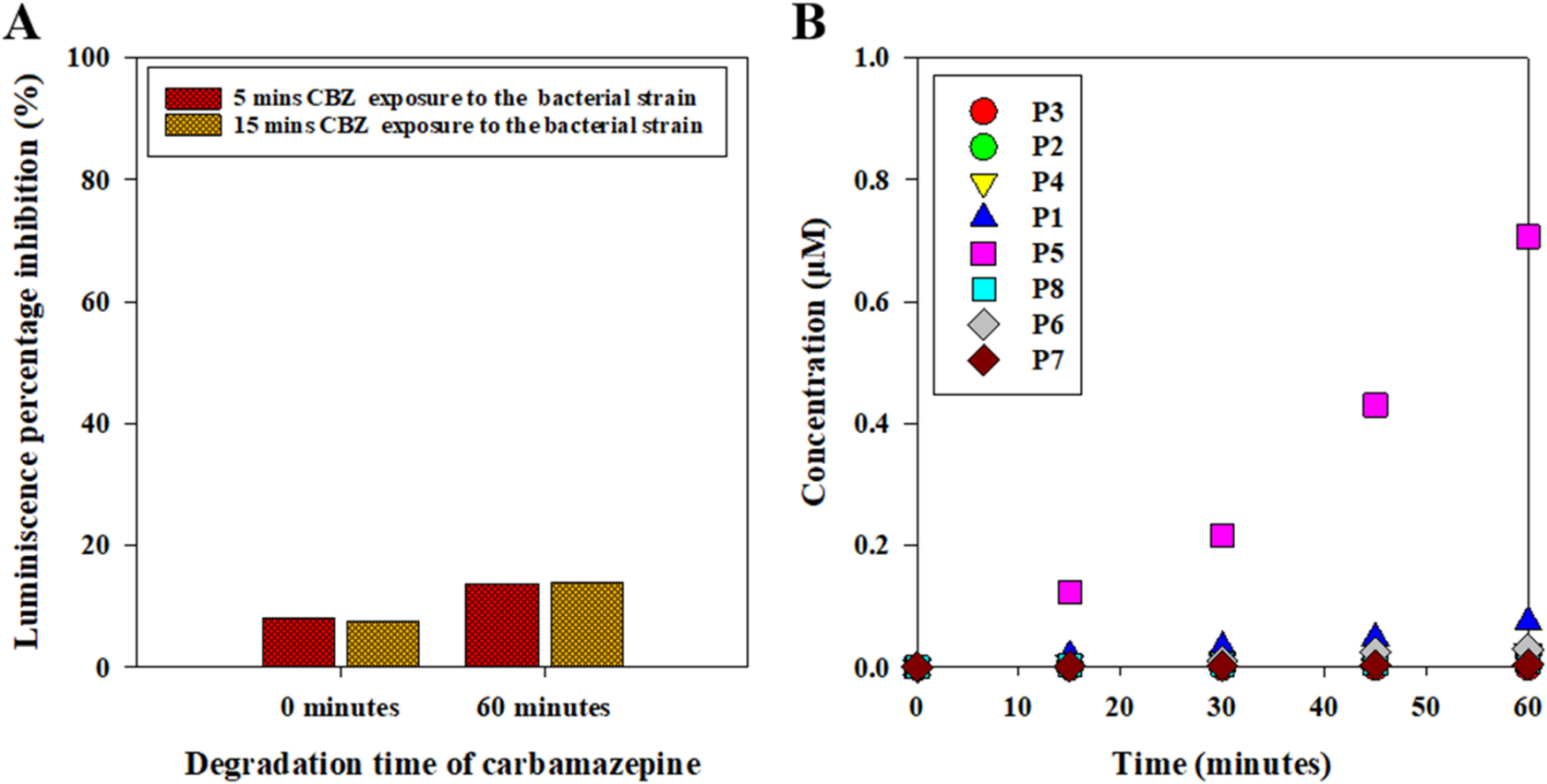
UV_305_/Cl degradation of 25 μM CBZ at pH 8 and the resulting (A) bioluminescence inhibition microtoxicity assay before and after 60 min of CBZ treatment, and (B) the evolution of CBZ transformation products (P1–P8) during the treatment.

### CBZ Degradation Byproducts and Proposed Pathway

Monitoring of CBZ degradation byproducts via mass spectrometry provided accurate mass-to-charge ratios (*m*/*z*) of several products. From these measurements, empirical formulas and molecular structures of oxidation products were determined; each species identified is shown in Table S7. A proposed reaction pathway for the transformation of CBZ in the UV_305_/Cl system is illustrated in Figure 9. The initial transformation of CBZ involves hydroxylation, yielding hydroxylated CBZ diols (P1). Subsequent ring contraction of P1 generates intermediates P2 and P3. In parallel, CBZ undergoes hydroxylation and chlorine substitution to form a chloroalcohol derivative (P4). Intramolecular rearrangement of P4, accompanied by HCl elimination, produces either epoxides or hydroxylated intermediates. These specific transformation reactions have been observed in previous CBZ degradation studies.^3,79^ Protonated epoxide intermediates derived from P4 can further hydrolyze to yield CBZ epoxide (P5). Chlorinated transformation products of P4 were confirmed through chlorine isotopic patterns, resulting in monochlorinated and dichlorinated species (P6 and P7, respectively).^3,111^ P7 undergoes heterocyclic ring oxidation, forming a new carbonyl group and producing intermediate P8. With progressive oxidative ring opening, the process ultimately generates low-molecular-weight chlorinated byproducts, including dichloroacetic acid (DCAA) and trichloroacetic acid (TCAA). Overall, these findings demonstrate that CBZ degradation under UV/Cl proceeds through a sequence of hydroxylation, chlorination, and ring-opening reactions, producing intermediates with moderate toxicity.

**Figure 9:**
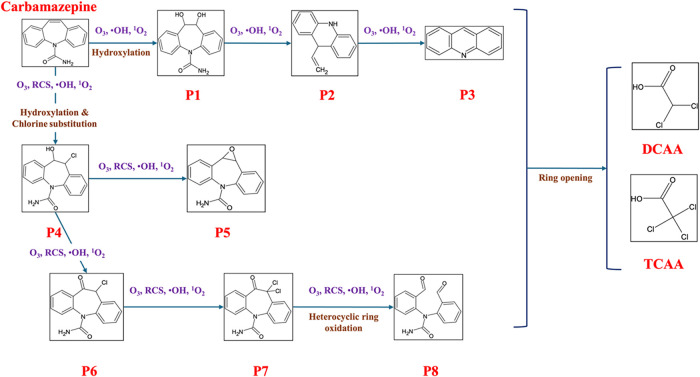
Proposed reaction pathways of CBZ degradation during UV_305_/Cl, at pH 8.

## Conclusions

The problem of unintended reactions between reactive species and DOM has been a persistent challenge in AOPs. However, UV/Cl, combined with newly available wavelength control (via UV-LEDs) offers a novel approach that may be particularly advantageous for tertiary wastewater treatment. Wavelength adjustments allow oxidant tuning to enhance contaminant degradation efficiency by minimizing undesired reactions with DOM. Here, a series of experiments highlight the ability to optimize CBZ removal across pHs and DOM isolates by shifting the irradiation wavelength. Drastic changes in CBZ degradation were observed under conditions that favored different reactive species distributions. Specifically, conditions promoting O_3_ formation caused faster CBZ removal compared to ^•^OH-driven pathways in the presence of DOM. The differential impact of various DOM isolates indicates that the organic composition of the water matrix significantly influences the AOP’s performance. CBZ degradation was least inhibited by colloids under O_3_-promoting conditions, likely because of ozone’s comparatively lower reactivity with the proteinaceous nature of the colloidal and SMP-like organics.^101^ The selectivity of O_3_ during treatment underscores the critical interplay between solution conditions and UV dosing parameters for the degradation of a targeted contaminant. The opportunity to drive the UV/Cl system with UV-LEDs provides unprecedented control over AOPs, because lamps can be assembled with multiple wavelengths available. In such a design, changes in influent pH, DOM content, or even dissolved oxygen can be accommodated by in real-time by a simple adjustment of irradiation wavelength to optimize ROS/RCS activity. The use of wavelengths longer than 254 nm garners additional efficiency gains, given that DOM absorption decreases significantly with increasing wavelength.

While further study is warranted, especially for potable water applications, the toxicity assays here do not indicate that the UV/Cl system is likely to yield higher toxicity than other AOPs. It may find excellent use as a tertiary polishing step for wastewater reclamation, with attention to transformation products. In sum, LED-driven chlorine photolysis attains a higher level of process control than conventional AOPs, with the intriguing opportunity of in situ O_3_ production as a selective oxidant. Conceivably, such a system could be appended to an extant chlorination system, allowing first for the conventional disinfection step and subsequently UV/Cl to double the utility of the chlorine dosage. Moreover, while the present work focuses on a scenario of tertiary wastewater treatment, the wavelength-tuning strategy demonstrated here is broadly applicable to drinking water systems as well, highlighting the potential of UV/Cl as a practical and scalable advanced oxidation platform.

## Supplementary Material



## Notes

Additional texts describing 3DEEM, quenching, and EPR analyses; tables containing corresponding categorization definitions, rate constants, calculated concentrations, and degradation byproduct molecules; and figures providing kinetic regression analyses for CBZ removal in several experimental or control conditions: pH variation, dark/irradiation wavelengths, and the presence of DOM isolates, reaction schema for spin trapping reactions, 3DEEM HA controls (PDF)
