# Supplementary material for: Advanced Chlorine Photolysis for Carbamazepine Degradation: Tuning Radicals for Dissolved Organics
Source: ACS ES T Water. 2026 Apr 14;6(5):3006–20. doi: 10.1021/acsestwater.5c01498 (PMC13162268; doi:10.1021/acsestwater.5c01498)
Supplement: Supplementary file 1 [file ew5c01498_si_001.pdf]

Supporting Information for Advanced Chlorine Photolysis for Carbamazepine Degradation: Tuning Radicals for Dissolved Organics

Caleb Osei-Appau[1], Madhusudan Kamat[1], Eddy Petit[2], Jeanne Le Beux[3], Benoit Teychené[4], Volodymyr V. Tarabara[5,6], François Zaviska[3], Geoffroy Lesage[3], Samuel D. Snow[1*]

[1]Department of Civil and Environmental Engineering, Louisiana State University, 3255 Patrick Taylor Hall, Baton Rouge, Louisiana 70803, United States.

[2]UAR Plateforme d'Analyses et Caractérisations (PAC) Chimie Balard Montpellier, Univ Montpellier, CNRS, ENSCM, Place Eugène Bataillon, 34095 Montpellier, France.

[3]IEM (Institut Européen des Membranes), UMR 5635 (CNRS-ENSCM-UM), Université de Montpellier, Place E. Bataillon, 34095, Montpellier, France.

[4]IC2MP (Institut de Chimie des Milieux et Matériaux de Poitiers), UMR CNRS 7285), Université de Poitiers, 1 rue Marcel Doré, 86073 Poitiers Cedex 9, France.

[5]Department of Civil and Environmental Engineering, Michigan State University, 428 S. Shaw Lane, East Lansing, Michigan 48824, United States.

[6]Center for European and Eurasian Studies, International Studies and Programs, Michigan State University, East Lansing, Michigan 48824, United States.

*Corresponding author: Email: ssnow@lsu.edu – Telephone: (+1) 225 578-8526

Address: Department of Civil and Environmental Engineering, Louisiana State University, 3255 Patrick Taylor Hall, Baton Rouge, Louisiana 70803, United States.

Content (11 pages): Text: S1-S2, Table: S1-S7, and Figures: S1-S10.

Text S1: 3DEEM Analyses

3DEEM spectra were collected using a Perkin-Elmer LS-55 spectrofluorometer following filtration of samples through a 2.0 µm filter. [1] Excitation and emission scan ranges were set between 200–500 nm and 280–600 nm, respectively. [1, 2] Measurements were performed at a scan speed of 1500 nm/min, with a 10 nm increment and slit widths of 10 nm for both excitation and emission. 3 All spectra were baseline-corrected using a Milli-Q water blank. [4] Based on the method described by Chen et al. (2003), [2] the fluorescence landscape was segmented into regions: Regions I and II represent aromatic protein-like substances (Ex: 200–250 nm / Em: 280–380 nm), Region III reflects fulvic acid-like substances (Ex: 200–250 nm / Em: 380–600 nm), Region IV corresponds to soluble microbial product-like proteins (Ex: 250–350 nm / Em: 280–380 nm), and Region V relates to humic acid-like compounds and their breakdown products (Ex: 380–600 nm / Em: 250–500 nm as shown in Table S1.

Text S2: Calculation of reactive species concentrations

The degradation of CBZ is assumed to proceed through bimolecular reactions with short-lived reactive species generated during chlorine photolysis, yielding a steady-state RS concentration. The pseudo-steady-state concentrations of reactive species (RS) were estimated according to the formulae below:

$r_{CBZ\cdot RS} = k_{CBZ\cdot RS}[CBZ][RS]_{ss}$,

$k_{obsCBZ\cdot RS} = k_{No\_quenchers} - k_{Quencher}$, and

$[RS]_{SS} = \left(\frac{k_{obs,CBZ\cdot RS}}{k_{CBZ\cdot RS}}\right)$,

where $r_{CBZ\cdot RS}$ is the rate at which CBZ is degraded via its reaction with a RS; $k_{CBZ.RS}$ is the second-order rate constant between a reactive species and CBZ ($M^{-1}s^{-1}$); $k_{obsCBZ\cdot RS}$ is the is the observed rate constant between a reactive species and CBZ (s^{-1}); $[RS]_{ss}$ is the calculated steady-state reactive species concentration (M); and [CBZ] is the initial CBZ concentration (M).

The pseudo–first-order rate constant of ozone was determined according to the formula:

$k_{obsCBZ.Ozone} = k_{CBZ\cdot Ozone}[Ozone]_{ss}$,

where $k_{obsCBZ\cdot Ozone}$ is the pseudo–first-order rate constant between O_3 and CBZ (s^{-1}); $[Ozone]_{ss}$ is the calculated steady-state concentration of O_3 (M); and $k_{CBZ\cdot Ozone}$ is the second-order rate constant between a O_3 and CBZ ($M^{-1}s^{-1}$).

Table S1. Categorization of fluorophore types in different regions of 3DEEM analysis. [2]

Region	Association	Excitation and Emission Range
I	Aromatic protein-like fluorophores, type I	Ex=200-250 nm/Em=280-330nm
II	Aromatic protein-like fluorophores, type II	Ex=200-250nm/Em=330-350nm
III	Fulvic acid-like fluorophores	Ex=200-250nm/Em=380-600nm
IV	SMP-like fluorophores	Ex=250-350nm/Em=280-380nm
V	Humic acid-like fluorophores	Ex=380-600nm/Em=250-500nm

Table S2. Observed rate constants (s^{-1}) of the radical adduct formation with RCS and ROS in the UV_{305}/Cl system at different pHs.

Radical Adduct	pH 3	pH 5	pH 8	pH 10
**BMPO-OH**	$1.86 \pm 0.003 \times 10^{-7}$	$1.20 \pm 0.003 \times 10^{-7}$	$9.54 \pm 0.003\times 10^{-8}$	$2.57 \pm 0.0004 \times 10^{-8}$
**DMPO-X**	$3.65 \pm 0.006 \times 10^{-7}$	$2.49 \pm 0.006 \times 10^{-7}$	$1.60 \pm 0.006 \times 10^{-7}$	$8.99 \pm 0.001 \times 10^{-8}$

Table S3. Molar concentrations of spin-trapped radical adducts of RCS and ROS after 60 minutes of UV_{305}/Cl at different pHs, calculated via the ESR Studio software.

Radical Adduct	pH 3	pH 5	pH 8	pH 10
**BMPO-OH**	1.12×10^{-5}	6.94×10^{-6}	5.42×10^{-6}	1.55×10^{-6}
**DMPO-X**	2.34×10^{-5}	1.75×10^{-5}	9.53×10^{-6}	5.41×10^{-6}

Table S4. The observed rate constants of carbamazepine degradation in the presence and absence of quenchers in the UV_{305}/Cl system at pH 8.

Quencher	k_{obs} (s^{-1})
No quenchers	1.933×10^{-4}
Tert-butyl alcohol	1.196×10^{-4}
Nitrobenzene	1.033×10^{-4}
Sodium benzoate	9.368×10^{-5}
Sodium azide	7.564×10^{-5}

Table S5. Reported second-order rate constants for the reactive species with carbamazepine.

Reactive species	Reported second-order rate constant for the reactive species with carbamazepine ($M^{-1}s^{-1}$)	References
•OH	4.6×10^9	[5]
Cl•	4.3×10^8	[6, 7]
$^1O_2$	5.0×10^4	[7, 8]
O_3	3.0×10^5	[9]

Table S6. Calculated steady-state concentrations of the reactive species in the UV_{305}/Cl system at pH 8.

Reactive species	Calculated [Reactive species]$_{ss}$ (M)	Reported [Reactive species]$_{ss}$ (M)	Reference
•OH	1.602×10^{-14}	10^{-14}	[10-12]
Cl•	2.317×10^{-13}	10^{-13}	[10-12]
$^1O_2$	2.353×10^{-9}	$10^{-14} – 10^{-12}$	[13, 14]
O_3	1.970×10^{-6}	10^{-8}	[12]

Table S7. List of primary by-products detected and used to assemble the proposed CBZ degradation pathways.

No	Product Number	Chemical Formula	Chemical Structure	$[M+H]^+$ or $[M-H]^-$ ions
1	Carbamazepine	$C_{15}H_{12}N_2O$	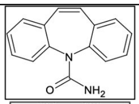	$[M+H]^+ = 237.1022$
2	P1	$C_{15}H_{14}N_2O_3$	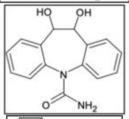	$[M+H]^+ = 271.1077$
3	P2	$C_{15}H_{13}N$	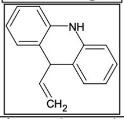	$[M+H]^+ = 208.1121$
4	P3	$C_{13}H_9N$	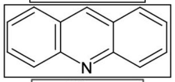	$[M+H]^+ = 180.0878$
5	P4	$C_{15}H_{13}ClN_2O_2$	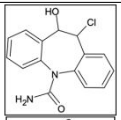	$[M+H]^+ = 289.0738$
6	P5	$C_{15}H_{12}N_2O_2$	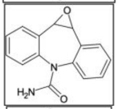	$[M+H]^+ = 253.0972$
7	P6	$C_{15}H_{11}ClN_2O_2$	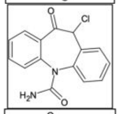	$[M+H]^+ = 287.0582$
8	P7	$C_{15}H_{10}Cl_2N_2O_2$	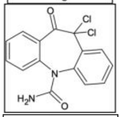	$[M+H]^+ = 321.0192$
9	P8	$C_{15}H_{12}N_2O_3$	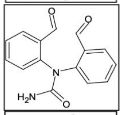	$[M+H]^+ = 269.0921$
10	DCAA	$C_2H_2Cl_2O_2$	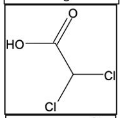	$[M-H]^- = 126.9359$
11	TCAA	$C_2HCl_3O_2$	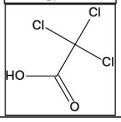	$[M-H]^- = 160.8969$

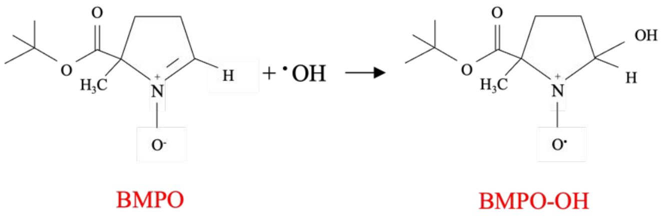

Fig. S1. Reaction scheme for •OH spin trapping by BMPO.

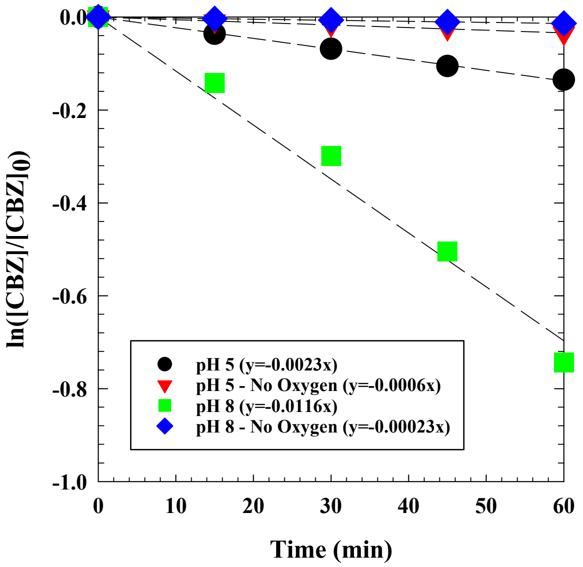

Fig. S2. Degradation kinetics of CBZ (25 μM) by UV_{305}/Cl using 5 mg-Cl_2/L at pH 8 and pH 5 in the presence and absence of oxygen.

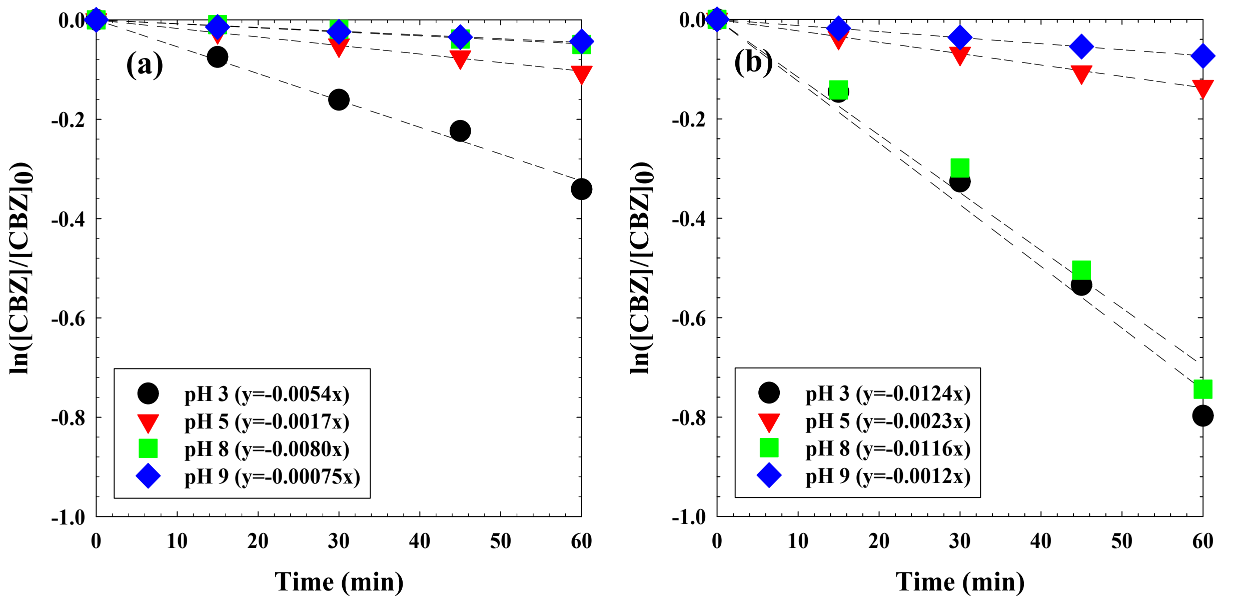

Fig. S3. Degradation kinetics of CBZ (25 μM) using 5 mg-Cl_2/L at varying pH values under (a) dark conditions and (b) UV_{305} irradiation.

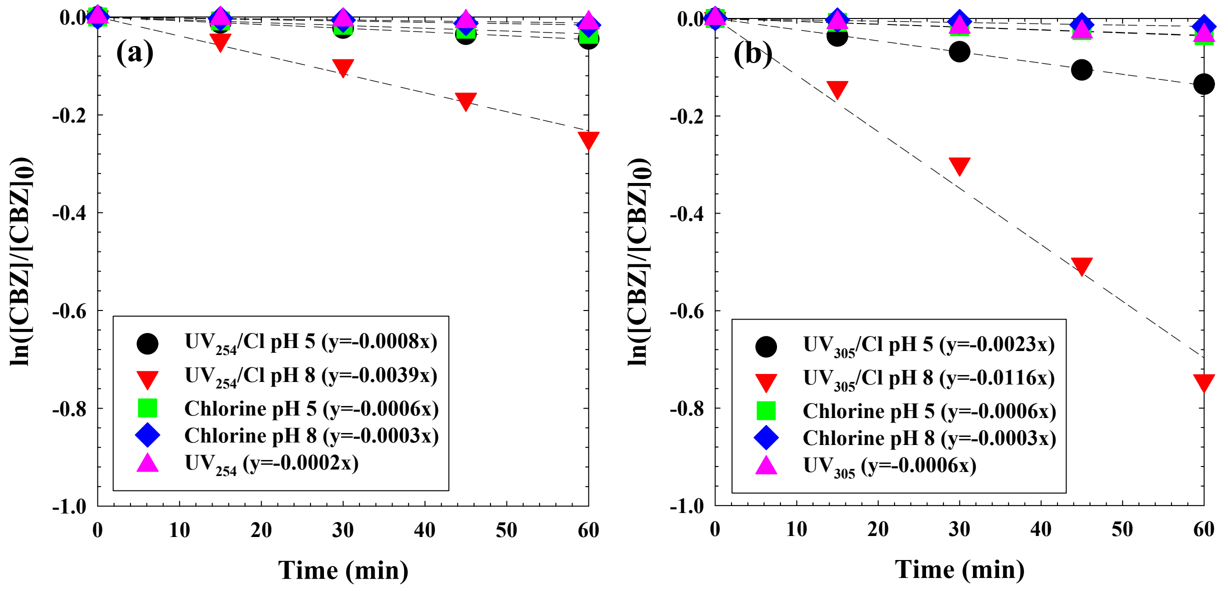

Fig. S4. Degradation kinetics of CBZ (25 μM) using 5 mg-Cl_2/L at varying pH values under (a) dark conditions and (b) UV_{305} irradiation.

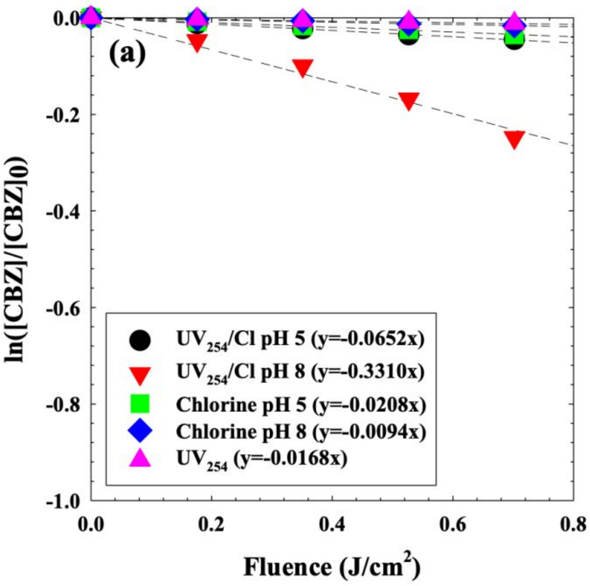

Fig. S5. Degradation of 25 μM CBZ as a function of fluence by 5 mg-Cl_2/L with (a) UV_{254}/Cl, free chlorine only (dark), or UV_{254} irradiation only; and (b) UV_{305}/Cl, free chlorine only (dark), or UV_{305} irradiation only.

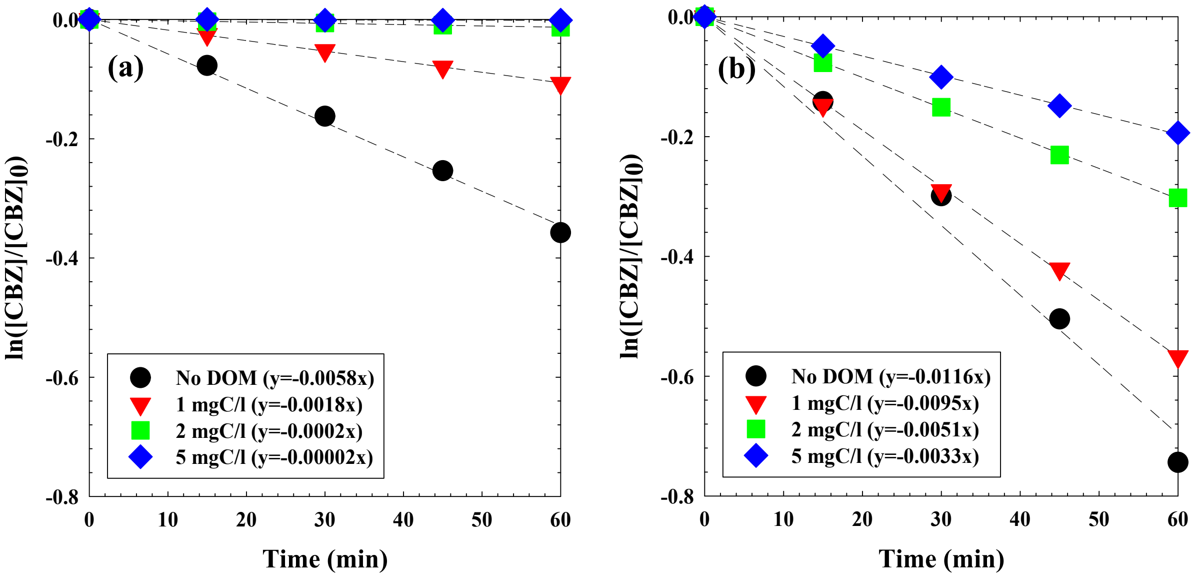

Fig. S6. Degradation kinetics of CBZ (25 μM) in the presence of 5 mg-Cl_2/L and varying concentrations of colloids under UV_{305}/Cl system at (a) pH 5 and (b) pH 8.

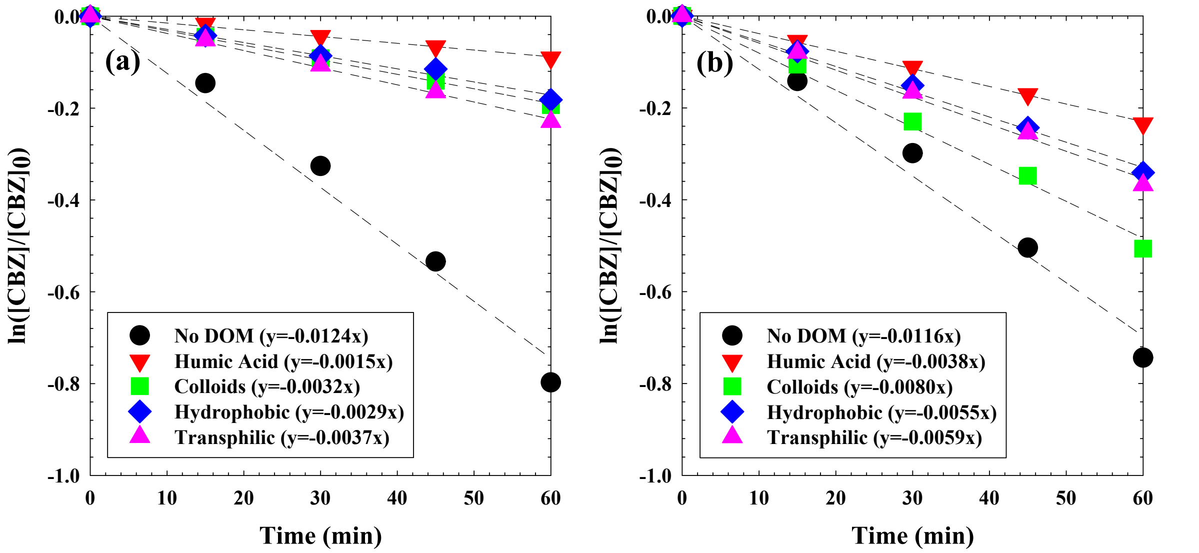

Fig. S7. Degradation kinetics of CBZ (25 µM) with 1 mg-C/L DOM fractions in the UV_{305}/Cl system using 5 mg-Cl_2/L at (a) pH 3 and (b) pH 8.

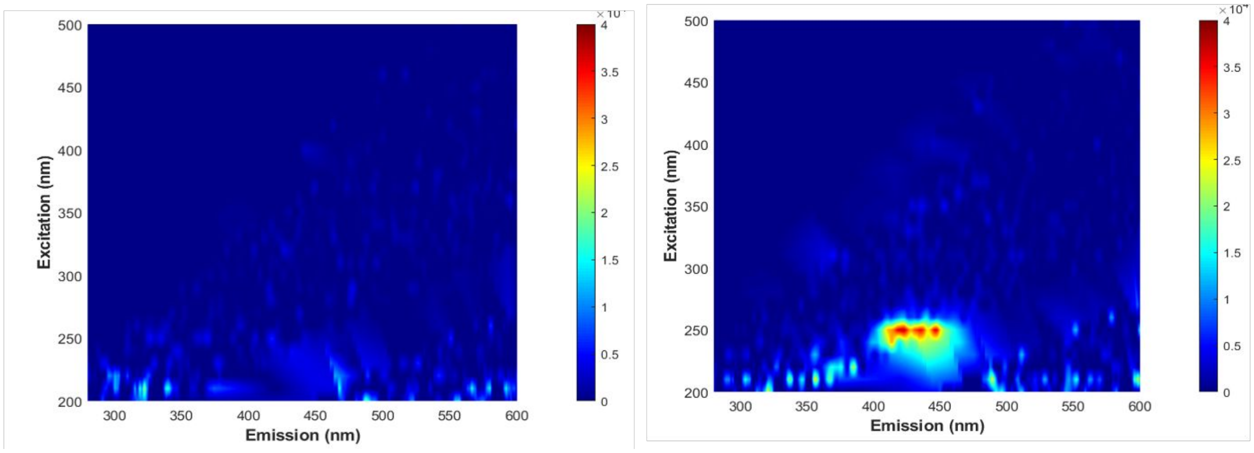

Fig. S8. 3DEEM spectra of humic acid a) before and b) after treatment with UV_{305}/Cl with 5 mg/L Cl_2 for 60 min.

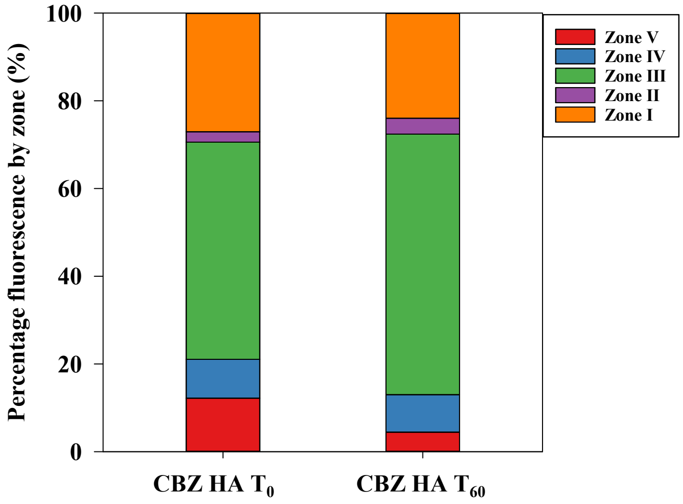

13

Fig. S9. Humic acid fluorescence percentage of exposure (at 0 and 60 minutes) to UV_{305}/Cl, at pH 8, using 5 mg-Cl_2/L. Regions are categorized as I) aromatic protein-like, type I; II) aromatic protein-like, type II; III) fulvic acid-like; IV) SMP-like; and V) humic acid-like fluorophores.

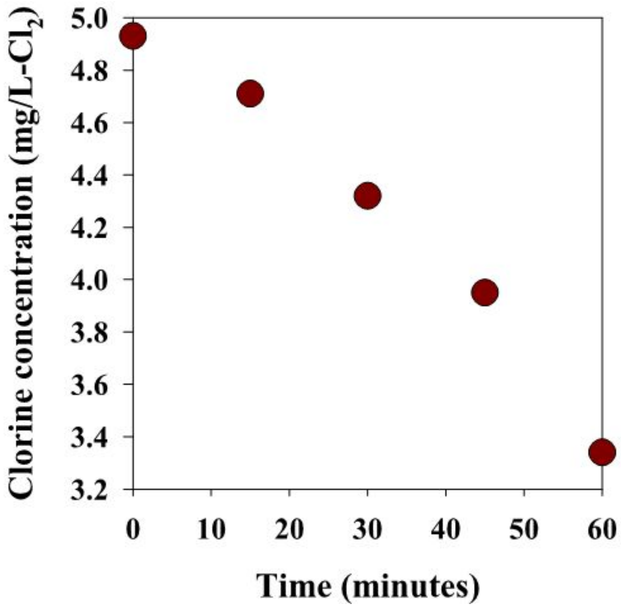

Fig. S10. Chlorine decay in a solution of 1 mg-C/L humic acid with UV_{305} at pH 8.

References

(1) Jacquin, C.; Teychene, B.; Lemee, L.; Lesage, G.; Heran, M. Characteristics and fouling behaviors of Dissolved Organic Matter fractions in a full-scale submerged membrane bioreactor for municipal wastewater treatment. *Biochem Eng J* **2018**, *132*, 169-181. DOI: 10.1016/j.bej.2017.12.016.

(2) Chen, W.; Westerhoff, P.; Leenheer, J. A.; Booksh, K. Fluorescence excitation-emission matrix regional integration to quantify spectra for dissolved organic matter. *Environ Sci Technol* **2003**, *37* (24), 5701-5710. DOI: 10.1021/es034354c.

(3) Azaïs, A.; Mendret, J.; Gassara, S.; Petit, E.; Deratani, A.; Brosillon, S. Nanofiltration for wastewater reuse: Counteractive effects of fouling and matrice on the rejection of pharmaceutical active compounds. *Sep Purif Technol* **2014**, *133*, 313-327. DOI: 10.1016/j.seppur.2014.07.007.

(4) Zepp, R. G.; Sheldon, W. M.; Moran, M. A. Dissolved organic fluorophores in southeastern US coastal waters: correction method for eliminating Rayleigh and Raman scattering peaks in excitation–emission matrices. *Mar Chem* **2004**, *89* (1-4), 15-36. DOI: 10.1016/j.marchem.2004.02.006.

(5) Xiao, R.; Ma, J.; Luo, Z.; Zeng, W.; Wei, Z.; Spinney, R.; Hu, W. P.; Dionysiou, D. D. Experimental and theoretical insight into hydroxyl and sulfate radicals-mediated degradation of carbamazepine. *Environmental Pollution* **2020**, *257*, 113498.

(6) Fang, Z.; Huang, R. F.; How, Z. T.; Jiang, B.; Chelme-Ayala, P.; Shi, Q.; Xu, C. M.; El-Din, M. G. Molecular transformation of dissolved organic matter in process water from oil and gas operation during UV/H_2O_2, UV/chlorine, and UV/persulfate processes. *Sci Total Environ* **2020**, *730*. DOI: 10.1016/j.scitotenv.2020.139072.

(7) Watts, M. J.; Linden, K. G. Chlorine photolysis and subsequent OH radical production during UV treatment of chlorinated water. *Water Res* **2007**, *41* (13), 2871-2878, Article. DOI: 10.1016/j.watres.2007.03.032.

(8) Liu, K.; Yu, J. C.; Dong, H.; Wu, J. C. S.; Hoffmann, M. R. Degradation and Mineralization of Carbamazepine Using an Electro-Fenton Reaction Catalyzed by Magnetite Nanoparticles Fixed on an Electrocatalytic Carbon Fiber Textile Cathode. *Environ Sci Technol* **2018**, *52* (21), 12667-12674. DOI: 10.1021/acs.est.8b03916.

(9) Psaltou, S.; Kaprara, E.; Mitrakas, M.; Zouboulis, A. Comparative study on heterogeneous and homogeneous catalytic ozonation efficiency in micropollutants' removal. *AQUA—Water Infrastructure, Ecosystems and Society* **2021**, *70* (8), 1121-1134.

(10) Peng, T.; Xu, C.; Yang, L.; Yang, B.; Cai, W. W.; Gu, F.; Ying, G. G. Kinetics and Mechanism of Degradation of Reactive Radical-Mediated Probe Compounds by the UV/Chlorine Process: Theoretical Calculation and Experimental Verification. *ACS Omega* **2022**, *7* (6), 5053-5063. DOI: 10.1021/acsomega.1c06001.

(11) Azad, A.; Iradukunda, J. C.; Men, Y.; Verdi, A.; Liu, H. Persulfate photolysis and limited irrigation of recycled wastewater for turfgrass growth: Accumulation of pharmaceutical and personal care products and physiological responses. *Water Res* **2024**, *262*, 122009. DOI: 10.1016/j.watres.2024.122009.

(12) Amichi, H.; Merouani, S.; Dehane, A.; Bouchoucha, H.; Hamdaoui, O. Photo (solar)-Activated Hypochlorite Treatment: Radicals Analysis Using a Validated Model and Assessment of Efficiency in Organic Pollutants Degradation. *Processes* **2024**, *12* (9), 1853.

(13) Ossola, R.; Jonsson, O. M.; Moor, K.; McNeill, K. Singlet Oxygen Quantum Yields in Environmental Waters. *Chem Rev* **2021**, *121* (7), 4100-4146. DOI: 10.1021/acs.chemrev.0c00781.

(14) Mackey, E.; Hofmann, R.; Festger, A.; Vanyo, C.; Moore, N.; Chen, T.; Wang, C.; Taylor-Edmonds, L.; Andrews, S. A. UV-chlorine advanced oxidation for potable water reuse: A review of the current state of the art and research needs. *Water Res X* **2023**, *19*, 100183. DOI: 10.1016/j.wroa.2023.100183.
